# SUMO conjugation to the pattern recognition receptor FLS2 triggers intracellular signalling in plant innate immunity

**DOI:** 10.1038/s41467-018-07696-8

**Published:** 2018-12-05

**Authors:** Beatriz Orosa, Gary Yates, Vivek Verma, Anjil K. Srivastava, Moumita Srivastava, Alberto Campanaro, Daniel De Vega, Alanna Fernandes, Cunjin Zhang, Jack Lee, Malcolm J. Bennett, Ari Sadanandom

**Affiliations:** 1 Department of Biosciences, Durham University, Stockton Road, Durham, DH1 3LE UK; 2 Plant & Crop Sciences, School of Biosciences, University of Nottingham, Leicestershire, LE12 5RD UK

## Abstract

Detection of conserved microbial patterns by host cell surface pattern recognition receptors (PRRs) activates innate immunity. The FLAGELLIN-SENSITIVE 2 (FLS2) receptor perceives bacterial flagellin and recruits another PRR, BAK1 and the cytoplasmic-kinase BIK1 to form an active co-receptor complex that initiates antibacterial immunity in *Arabidopsis*. Molecular mechanisms that transmit flagellin perception from the plasma-membrane FLS2-associated receptor complex to intracellular events are less well understood. Here, we show that flagellin induces the conjugation of the SMALL UBIQUITIN-LIKE MODIFIER (SUMO) protein to FLS2 to trigger release of BIK1. Disruption of FLS2 SUMOylation can abolish immune responses, resulting in susceptibility to bacterial pathogens in *Arabidopsis*. We also identify the molecular machinery that regulates FLS2 SUMOylation and demonstrate a role for the deSUMOylating enzyme, Desi3a in innate immunity. Flagellin induces the degradation of Desi3a and enhances FLS2 SUMOylation to promote BIK1 dissociation and trigger intracellular immune signalling.

## Abstract (web summary)

The plant FLS2 receptor initiates bacterial immunity in response to flagellin. Here the authors show that SUMO conjugates to FLS2 in response to flagellin promoting downstream signalling events while Desi3A, an FLS2 deSUMOylating enzyme, is degraded to enhance immune responses.

## Introduction

The first critical step in innate immune responses is the perception of pathogens via microbial- or pathogen-associated molecular patterns (MAMPs or PAMPs)^1–6^. Plants utilize plasma membrane-localized pattern recognition receptors (PRRs) to perceive PAMPs. A well-studied PRR in plants is the Arabidopsis leucine-rich repeat (LRR) receptor kinase FLAGELLIN SENSING 2 (FLS2). FLS2 recognizes a conserved N-terminal 22-amino acid sequence (flg22) of bacterial flagellin^7^ as a PAMP initiating pattern-triggered immunity (PTI)^8–10^. In addition to an extracellular LRR receptor domain, FLS2 also contains a transmembrane domain and a cytoplasmic serine/threonine kinase domain qualifying it as an LRR-receptor kinase^11^. Under uninfected conditions, FLS2 is complexed with an intracellular kinase BIK1 (Botrytis-induced kinase 1)^12–14^. Upon flg22 perception during bacterial infection, FLS2 recruits another LRR receptor-like kinase (RLK) brassinosteroid insensitive 1-associated kinase 1 (BAK1)^15,16^. Recent advances showed that BAK1 is a coreceptor of flg22 and intermolecular interaction between the LRR domains of FLS2 and BAK1 initiate the activation of the PRR complex. BIK1 is directly phosphorylated by BAK1^13,14^ and dissociates from FLS2 in a BAK1 dependent manner to activate downstream signalling components. This includes activation of mitogen-activated protein kinases (MAPKs) and respiratory burst oxidase homologue protein D (RbohD) to generate a burst of reactive oxygen species (ROS)^12,17^ to trigger immune signalling, which potentially halts the pathogen before it establishes within the plant host.

Therefore, in FLS2-mediated immune signalling, dissociation of BIK1 from FLS2 complex is a crucial and rate-limiting step^18^. Although, a magnitude of studies have provided great insight into the mechanism of FLS2 signalling during the past decade, a major crevice in our understanding lies in the early molecular events that regulate the FLS2-BIK1 dissociation^12–14,17,19,20^. In this study, we reveal that the mechanism governing FLS2-BIK1 dissociation relies on SUMOylation of FLS2.

SUMOylation is emerging as a key PTM in plants. SUMO conjugation to its cognate target relies on an enzyme cascade akin to ubiquitination for activation and ligation of the SUMO polypeptide to lysine residues in target proteins. This process is reversed by deconjugating SUMO protease enzymes. Regulated SUMO deconjugation of target protein provides rapid flexibility to protein interaction networks. Our data sheds light on how SUMO plays a critical regulatory role in FLS2-mediated innate immune response. In this study we demonstrate that SUMOylation of FLS2 is enhanced upon flagellin perception and this modification facilitates its dissociation from BIK1. We identify a class of membrane-localized SUMO proteases that are able to target the FLS2 receptor for deSUMOylation to regulate immune signalling.

## Results

### FLS2-mediated immune responses require SUMOylation

**Fig. 1:**
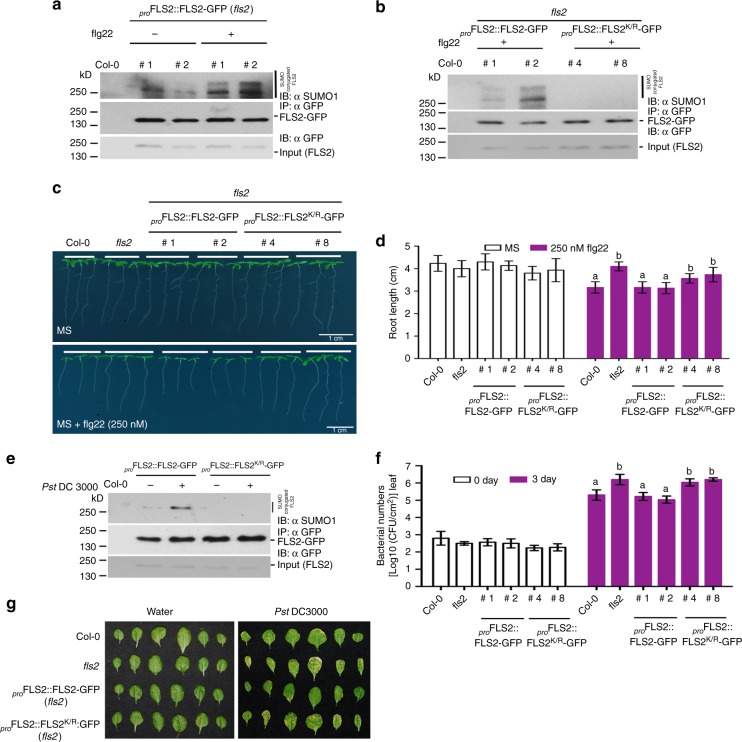
SUMO conjugation to FLS2 is flg22 dependant and required for innate immunity in Arabidopsis. **a** FLS2 is rapidly SUMOylated after flg22 treatment. Immunoprecipitation (IP: αGFP) was carried out with anti-GFP beads from total protein derived from two independent transgenic lines expressing _*pro*_*FLS2::FLS2-GFP* (*fls2*) treated either with MgCl_2_ or 1 µM flg22 for 10 min. Immunoblots were probed with anti-GFP (IB: αGFP) or anti-SUMO1/2 (IB: αSUMO1) antibodies. Col-0 was used as a negative control. **b** Mutation of Lysine 1120 to Arginine in FLS2 (FLS2^K/R^) abolishes SUMOylation. Immunoprecipitation was performed as described in (**a**). Two independent transgenic lines were analysed per genotype. **c** FLS2^K/R^-GFP transgenics are insensitive to flg22-mediated growth inhibition. A representative image of Col-0, *fls2* and two independent transgenic lines each of FLS2-GFP and FLS2^K/R^-GFP seedlings in 1/2MS or 1/2MS with 250 nM flg22. Scale bar = 1 cm. **d** Quantification of root growth inhibition for Col-0, *fls2*, FLS2-GFP and FLS2^K/R^-GFP transgenic lines. Four-day-old seedlings of the different genotypes were transferred to 1/2MS or 1/2MS with 250 nM flg22 and grown for additional 6 days before root lengths were measured. Data presented are means ± SE from at least 25 individual seedlings per genotype (Bars with different letters were significantly different from others; *P* < 0.05; two-way ANOVA with post hoc Tukey test). **e** FLS2 is rapidly SUMOylated after virulent *Pst* DC3000 infection. Four-week-old transgenic lines expressing FLS2-GFP (#2) or FLS2^K/R^-GFP (#4) proteins were dipped in *Pst*. Total protein was extracted after 4 h and immunoprecipitated (IP: αGFP) as described in (**a**). **f** FLS2 SUMOylation is required for immunity against *P. syringae*. Four-week-old rosettes of Col-0, *fls2*, FLS2-GFP, FLS2^K/R^-GFP transgenic plants were dipped in liquid cultures of *Pst*. and colony-forming units (CFUs) were counted at day 0 and 3 days post-infection (dpi). Values are means ± SE from three independent biological replicates with 8–10 plants each replicate (*N* = 3; bars with different letters were significantly different from others; *P* < 0.05; two-way ANOVA with post hoc Tukey test). **g** FLS2^K/R^-GFP transgenics are more susceptible to *Pst* infection. Image of leaves from rosettes infected with *Pst*. in (**f**) were photographed five dpi

In a proteomic screen using transgenic plants expressing streptavidin tagged SUMO1 to identify SUMOylated proteins during flg22-triggered immune responses, we identified peptide fragments that match FLS2, suggesting that this receptor might be a potential target for SUMO conjugation (Supplementary Table 1). To ascertain if FLS2 is indeed SUMOylated we immunopurified FLS2-GFP in the absence and presence of flg22 from transgenic plants expressing _*pro*_*FLS2::FLS2*-GFP in *fls2* mutant background. Immunoblot experiments with anti-SUMO1 (*At*SUMO1) specific antibodies^21^ revealed that FLS2-GFP was significantly conjugated with SUMO1 after flg22 treatment, as compared to SUMO conjugation before flg22 treatment (Fig. 1a and Supplementary Fig. 20).

To understand the direct role of SUMOylation of FLS2-mediated immune signalling, we identified Lysine 1120 in the c-terminal cytoplasmic domain of FLS2 as a potential SUMO site. Strikingly, this SUMOylation site lysine residue in the cytoplasmic domain is conserved in FLS2 from six different members of Brassicaceae family and also with FLS2 from *Arabidopsis lyrata* and *Arabidopsis halleri* (Supplementary Fig. 1) suggesting an important regulatory role for SUMO in immune signalling.

To ascertain if SUMOylation is a factor in FLS2 triggered immune responses, we mutated the SUMO site Lysine 1120 in FLS2 to an Arginine residue where the acceptor side chain amine group can no longer be conjugated to SUMO and generated transgenic _*pro*_*FLS2::FLS2*^*K/R*^*-GFP* in *fls2* mutant background. In vivo SUMO immunoprecipitation assays indicated that when compared to wild-type FLS2 controls there was no detectable SUMOylation of FLS2^K/R^ mutant protein after flg22 treatment (Fig. 1b and Supplementary Fig. 20) demonstrating that this conserved lysine is the major site for SUMO1 attachment to FLS2. Transcript and protein expression analysis indicated that these were comparable between *FLS2-GFP* and *FLS2*^*K/R*^*-GFP* expressing transgenic plants (Supplementary Fig. 2a, b). Furthermore, we also found that the lack of SUMO conjugation to FLS2^K/R^-GFP neither correlated with anomalies in sub-cellular localization (Supplementary Fig. 3), nor with protein turnover rate differences between FLS2 and FLS2^K/R^ (Supplementary Fig. 4 and Supplementary Fig. 24).

Arabidopsis seedlings grown on agar plates supplemented with flg22 exhibit sensitivity to this peptide via root growth inhibition^15^. Exogenous flg22 treatment caused significant root growth inhibition in Col-0 and transgenic lines expressing wild-type FLS2-GFP; however, this effect was significantly reduced in transgenic lines expressing non-SUMOylatable *FLS2*^*K/R*^*-GFP* plants (Fig. 1c, d). Indeed growth of seedlings expressing non-SUMOylatable FLS2 mutants was not significantly different from *fls2* knockout mutant plants implying that the *FLS2*^*K/R*^ non-SUMOylatable mutant was not able to sense or respond to flagellin (Fig. 1c, d). This was further substantiated by transcript analysis of immunity related genes *FRK1, WRKY30* and *MYB51* upon flg22 treatment, where FLS2^K/R^ plants exhibited at least two-fold reduction of gene expression compared to plants expressing *FLS2-GFP* demonstrating that *FLS2*^*K/R*^*-GFP* non-SUMO mutant plants were impaired in perceiving flagellin or are less effective in triggering downstream immune signalling (Supplementary Fig. 5).

Detection of flagellin by FLS2 leads to the activation of PTI restricting bacterial growth, whilst *fls2* mutant plants fail to mount an effective PTI against the virulent bacterial pathogen bacteria *Pseudomonas syringae pv. tomato* (*Pst*) resulting in increased bacterial colonisation and disease development^9^. Hence, we investigated the SUMOylation status of FLS2 after *Pst*. infection. Strikingly, immunoprecipitation assays indicated that FLS2-GFP was SUMOylated after infection with virulent *Pst*, while there was no detectable SUMOylation of FLS2^K/R^-GFP (Fig. 1e and Supplementary Fig. 20). Since FLS2^K/R^ plants were unable to trigger efficient immune signalling, we wanted to ascertain whether *FLS2*^*K/R*^ plants are more susceptible to *Pst* Pathogen infection assays with *Pst* indicated that *FLS2*^*K/R*^ plants were more susceptible with increased bacterial growth and developed severe disease symptoms reminiscent of the *fls2* mutant (Fig. 1f, g). Indeed the levels of bacterial growth in FLS2^K/R^ plants were comparable to that in *fls2* mutants indicating that SUMOylation is vital for FLS2 dependent antibacterial immunity in Arabidopsis.

### BIK1 release from the PRR complex requires FLS2 SUMOylation

**Fig. 2:**
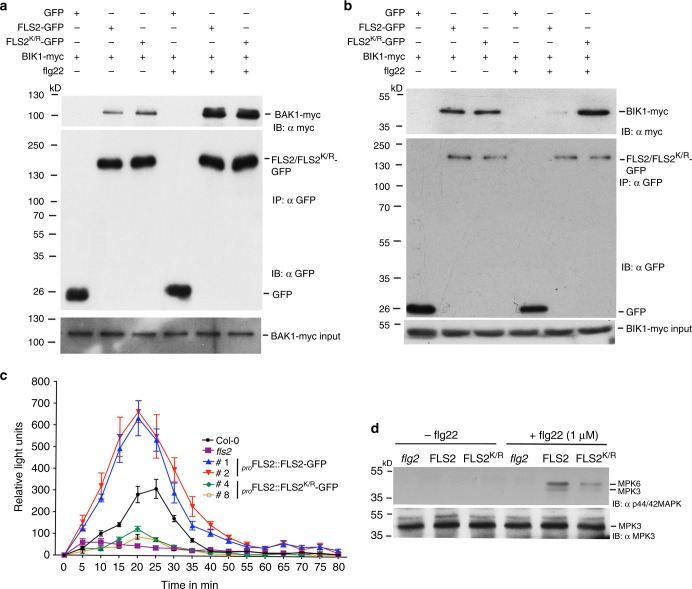
SUMOylation is required for BIK kinase release from activated FLS2-BAK1 immune complex to initiate immune signalling. **a** FLS2-GFP and FLS2^K/R^–GFP interacts with BAK1-myc in a flagellin dependant manner. *Nicotiana benthamiana* leaves transiently expressing FLS2-GFP or FLS2^K/R^-GFP with BAK1-myc were treated with MgCl_2_ or 1 µM flg22 for 10 min. Subsequently, total protein was subjected to immunoprecipitation with anti-GFP immunoaffinity beads (IP: αGFP) followed by immunoblot analysis with anti-myc (IB: αmyc) antibodies to detect BAK1-myc and anti-GFP (IB: αGFP) antibodies to detect FLS2-GFP. BAK1 protein levels in all samples were determined by probing with anti-myc antibodies to detect BAK1-myc in total protein extracts (BAK1-myc input). GFP was used as a negative control. **b** Non-SUMOylatable FLS2^K/R^-GFP remains bound to BIK1 after flagellin treatment. FLS2-GFP and FLS2^K/R^-GFP were transiently expressed with BIK1-myc in *N. benthamiana* and coimmunoprecipitation and immunoblotting was done as described in (**a**) except that BIK1 was analysed by antibodies against c-myc. Total protein of all samples was probed with anti-myc antibody to determine BIK1 protein levels (BIK1-myc input). GFP was used as a negative control. **c** SUMOylation of FLS2 is required for the induction of immune related ROS burst. Leaf discs from three-week-old plants of Col-0, *fls2*, and two transgenic lines each of FLS2-GFP, FLS2^K/R^-GFP were treated with 1 µM of flg22 for 10 min and ROS burst detected. Results shown are average ± SE (*n* = 3). **d** Non-SUMOylatable FLS2^K/R^-GFP plants exhibit significantly reduced levels of MAPK activation. Different genotypes indicated were treated with water or 1 µM of flg22 for 10 min and total protein were extracted after treatment. Immunoblots were probed with anti-p44/42 MAPK antibodies to detect activated MPK3 and 6 (upper panel); the lower panel was immunoblotted with anti-MPK3 antibody to ensure equal protein loading

The failure of FLS2^K/R^-GFP transgenic lines to elicit an effective immune response prompted us to determine if FLS2^K/R^ is impaired in recruiting BAK1 or subsequent signalling steps upon flg22 perception. To test BAK1 recruitment to the FLS2-BAK1 PRR complex, we immunoprecipitated FLS2-GFP or FLS2^K/R^-GFP in the absence or presence of flg22 from *Nicotiana benthamiana* co-expressing FLS2 proteins with BAK1-myc. Immunoblots (IB: αmyc) suggested that there was no significant difference in BAK1 recruitment by FLS2-GFP or FLS2^K/R^-GFP proteins (Fig. 2a and Supplementary Fig. 21). Similar results were obtained when BAK1-myc was immunopurified and the immunoprecipitate was probed for GFP-tagged FLS2 and FLS2^K/R^ proteins (Supplementary Fig. 6a–c). Furthermore, we ascertained if impaired immune responses in FLS2^K/R^-GFP are due to its inability to be phosphorylated upon flagellin perception. No discernable difference was observed in the trans-phosphorylation status of FLS2-GFP and FLS2^K/R^-GFP when the respective transgenic seedlings were treated with flg22 and FLS2- or FLS2^K/R^-GFP immunoprecipitates were probed with anti-phosphotyrosine antibody (Supplementary Fig. 7a and Supplementary Fig. 25). We, also, did not observe any difference in the autophosphorylation activity of FLS2 and FLS2^K/R11^. This was determined by incubating recombinantly expressed GST-tagged kinase domain of FLS2 (GST-FLS2^KD^; 838–1173) and FLS2^KD_K/R^ proteins with ATP and subsequently, subjecting the protein samples to Phos-tag gel analysis. The immunoblot analysis (IB: αGST) showed comparable levels of phosphorylation for both proteins (Supplementary Fig. 7b and Supplementary Fig. 25). This evidence further validated that SUMOylation has no role to play in auto- or trans-phosphorylation of FLS2.

Having confirmed that FLS2^K/R^ was neither defective in BAK1 recruitment nor in auto- and trans-phosphorylation activities, we screened for anomalies in the subsequent signalling step, i.e. release of BIK1 from FLS2-BAK1 complex after flagellin perception. Coimmunoprecipitation assays, in the absence and presence of flg22, from *N. benthamiana* leaves transiently expressing FLS2-GFP or FLS2^K/R^-GFP with BIK1-myc showed that both proteins precipitated with comparable levels of BIK1-myc in the absence of flg22 (Fig. 2b and Supplementary Fig. 21). However, in the presence of flg22, FLS2^K/R^ co-purified with significantly higher amounts of BIK1-myc in comparison to FLS2-GFP (Fig. 2b). This data indicated that the dissociation of BIK1 from the FLS2^K/R^-BAK1 immune complex upon flagellin perception is impaired (Fig. 2b). This suggests that SUMOylation of FLS2 is obligatory for BIK1 release from the FLS2-BAK1 complex, thereby suggesting a mechanism for BIK1 dissociation that relies on SUMOylation of FLS2.

To support the evidence that BIK1 is not released to trigger immune signalling from activated FLS2^K/R^-BAK1 immune complex, we monitored Arabidopsis FLS2-GFP and FLS2^K/R^–GFP transgenic lines for their ability to trigger an oxidative burst in response to flg22. We observed that, like *fls2* mutant, *FLS2*^*K/R*^ plants fail to trigger an oxidative burst, suggesting that SUMOylation of the FLS2 immune receptor is a prerequisite for pathogen induced oxidative burst, which is a hallmark of innate immunity in higher eukaryotes (Fig. 2c).

The dissociation of the BIK1 kinase is a major step in the generation of downstream immune signalling which includes induction of mitogen-activated protein kinase (MAPK) cascades that lead to multiple responses such as hormone biosynthesis and the activation of immune associated gene expression^5,22,23^. The activation of the MAPK cascade can be monitored by antibodies that detect the accumulation of phosphorylated MPK6 and MPK3^23^. Immunoblotting of protein extracts from *FLS2* and *FLS2*^*K/R*^ and mutant *fls2* plants with these antibodies indicates significant activation of MPK6 and MPK3 within 10 min of flg22 treatment in seedlings expressing only FLS2-GFP. However, this response was not observed in the *fls2* mutant and significantly reduced in the non-SUMOylatable *FLS2*^*K/R*^ lines, suggesting that SUMOylation of the FLS2 receptor is a major requirement for flg22-mediated activation of the MAPK protein kinase cascade (Fig. 2d and Supplementary Fig. 21). This data further supports our hypothesis that SUMOylation of the FLS2 receptor is critical for BIK1 release from the plasma membrane associated FLS2-BAK1 immune complex to initiate intracellular immune signalling.

### Identifying the SUMO protease that deSUMOylates FLS2

**Fig. 3:**
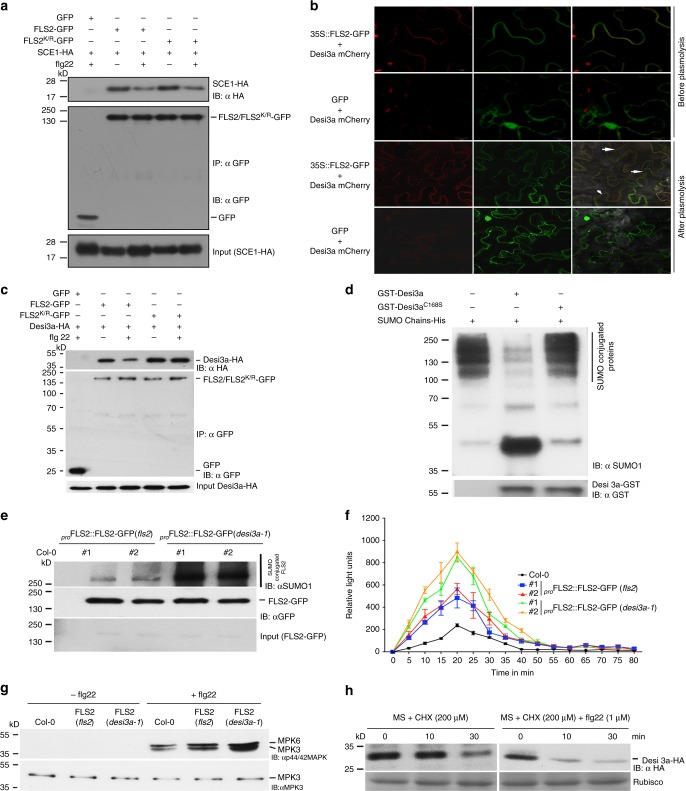
Identification of SUMO components controlling FLS2-mediated immune signalling. **a** SCE1 interacts with FLS2. *Nicotiana benthamiana* leaves transiently expressing FLS2-GFP/FLS2^K/R^-GFP with SCE1-HA were treated with MgCl_2_ or 1 µM flg22 (10 min). Immunoprecipitation was done with anti-GFP beads (IP: αGFP) and immunoblotted with anti-HA (IB: αHA) for SCE1-HA and anti-GFP (IB: αGFP) for GFP/GFP-fusion proteins. Total protein extracts were probed with anti-HA antibody (SCE1-HA input) for equal SCE1. **b** Desi3a co-localizes with FLS2-GFP to plasma membrane. *N. benthamiana* leaves co-infiltrated with mCherry-Desi3a and FLS2-GFP or GFP were detected for fluorescence after 3 days. Before imaging, cells were plasmolysed with 1 M NaCl (1 h). Images were obtained using confocal laser scanning microscope Carl Zeiss 880. Scale bar = 10 µm. Arrows indicate overlap of mCherry-Desi3a and FLS2-GFP signals. **c** FLS2 interacts with Desi3a. Coimmunoprecipitation from *N. benthamiana* leaves expressing FLS2-GFP/FLS2^K/R^-GFP or GFP with Desi3a-HA were performed. Total proteins were probed with anti-HA antibody for equal Desi3a (Desi3a-HA input). **d** Desi3a is a *bona fide* SUMO protease. High molecular conjugates of His-SUMO1 chains were incubated with GST-Desi3a (Desi3a^WT^) or Desi3a^C168S^ at 30 °C and subsequently immunoblotted with anti-SUMO1 (IB: αSUMO1) to detect SUMO chains (upper panel), and with anti-GST to detect GST-fusion proteins (lower panel). **e** FLS2 is hyper-SUMOylated in *desi3a-1*. Immunoprecipitates (IP: αGFP) from transgenic lines expressing *proFLS2::FLS-GFP* in *fls2* or *desi3a-1* were probed with anti-GFP (IB: αGFP) or anti-SUMO1/2 (IB: αSUMO1) antibodies. **f** HyperSUMOylation of FLS2 enhances ROS burst. Leaf discs from three-week-old plants of Col-0, FLS2-GFP (*fls2*), FLS2-GFP (*desi3a-1*) transgenic lines were treated with 1 µM flg22 (10 min) and ROS burst detected. Results shown are average ± SE (*n* = 3). **g** HyperSUMOylation of FLS2 increases MAPK activation. Col-0, FLS2-GFP (*fls2*) and FLS2-GFP (*desi3a-1*) transgenic lines were treated with water or 1 µM flg22 (10 min) and total proteins were probed with anti-p44/42 MAPK antibody to detect activated MPK3/6 (upper panel) and with anti-MPK3 antibody for equal protein loading. **h** flg22 degrades Desi3a. 10-day-old seedlings of *35S::Desi3a-HA* (*desi3a-1*) transgenic lines were treated with 200 μM cycloheximide or a combination of 200 μM cycloheximide + 1 μM flg22 and total proteins extracted at indicated time-points were immunobloted with anti-HA (IB: αHA) antibody

The SUMO machinery components that mediate the post-translational modification of the FLS2 immune receptor are likely to play a major role in innate immunity. SUMO proteins are processed to their mature form by SUMO proteases that cleave the C-terminal tail from the precursor, exposing the site where target attachment occurs in a series of enzymatic reactions very similar to ubiquitination, that includes activation, conjugation and ligation^21,24^. Despite the obvious similarity between SUMOylation and ubiquitination, the SUMO system in plants has an unusually low number of E3s compared to ubiquitination, with only two SUMO E3 ligases, AtHPY2 and AtSIZ1, encoded by the *Arabidopsis* genome^25^ whereas hundreds of ubiquitin E3s have been identified. How the SUMO system maintains target specificity within this context is still unknown. SUMO E3s so far identified reside within the nucleus^26^ while FLS2 is targeted to plasma membranes^27^. However, evidence has shown that the SUMO E2 conjugating enzyme is capable of directly transferring SUMO onto target residues^28^. The plant SUMO E2 (SCE1) is localized throughout the cell and immunoprecipitation assays with Agrobacterium-mediated transient assays in *N. benthamiana* indicated that SUMO E2, SCE1, interacts with FLS2 suggesting that SUMO E2 can directly add SUMO to FLS2 (Fig. 3a). Furthermore, the interaction of FLS2 with SUMO E2 was significantly reduced in the presence of flg22, suggesting enhanced dissociation of FLS2 from the FLS2-SCE1 complex after SUMO conjugation upon flagellin treatment.

Subsequently, we tested if SCE1 is able to SUMOylate FLS2 independently of any SUMO E3 ligase. To this end, we recombinantly expressed and purified GST-FLS2^KD^ from an *E. coli* system possessing reconstituted SUMO machinery barring SUMO E3^29^. Immunoblots from this SUMO reconstituted system probed with an anti-SUMO1 antibody (IB: αSUMO1) showed that FLS2 is poly-SUMOylated and SUMO E2 alone can directly add SUMO to FLS2. Mutated non-conjugatable *At*SUMO (AA) was used as a control where there was no ploySUMOylation of GST-FLS2^KD^ was observed (Supplementary Fig. 8).

SUMO proteases possess de-conjugation activity capable of cleaving SUMO from target proteins, providing reversibility and flexibility to the pathway^24^. These proteases play critical roles in maintaining the equilibrium in SUMO signalling^30^. Discreet subclasses of SUMO proteases influence specific aspects of plant development and responses to the environment with limited functional overlap indicating that the all-important substrate specificity within the plant SUMO system may be mediated by these DeSUMOylating proteases^30^. SUMO proteases belong to the cysteine protease family of proteolytic enzymes. These, often specialised proteases, are present in eukaryotes including plants with many residing within a species. Within the Arabidopsis proteome there are at least seven SUMO proteases classed as ubiquitin-like proteases (ULPs) but they are, predominantly, localised to the nucleus of plant cells^31–33^. Since FLS2 is membrane localised, we explored the possibility of a novel class of membrane localised SUMO proteases. Previously, a new class of SUMO proteases was identified in mammals, which contain a characteristic DeSUMOylating isopeptidase (Desi) domain^34,35^. This class of SUMO proteases belongs to the C97^34^ superfamily of cysteine proteases and was shown to be localized both inside and outside the nucleus. Therefore, we searched for Desi SUMO protease homologues in Arabidopsis. The amino acid sequence of the conserved catalytic domain of Human Desi-1 was used to search against the Arabidopsis proteome, enabling us to identify eight putative Desi proteins (Supplementary Fig. 9). It is noteworthy that proteins encoded by At1g80690 and At4g31980 were also identified during the search; however, they lacked key amino acid residues in their catalytic domain^34^ and hence, not likely to be bona fide SUMO proteases. Therefore, they were not included for any further analysis.

To date there is limited data on the cellular function of the Desi SUMO proteases and only one substrate has so far been described^35^. Of the eight Desi SUMO proteases we identified a protein encoded by the gene at the locus *AT1G47740* (will now on be referred to as *Desi3a*) to be localised in the membrane (Supplementary Fig. 10). Therefore, we investigated if FLS2 and Desi3a co-localize to the cell membrane by transiently co-expressing FLS2-GFP and Desi3a-mCherry in *N. benthamiana*. Agro-infiltrated leaf samples were plasmolysed with 1 M NaCl for 1 h to separate the vacuole from the cell membrane. Confocal images indicated that fluorescent signals originating from both fluorophores emanated from cell membranes suggesting that both FLS2-GFP and Desi3a-mCherry co-localize to the plasma-membrane (Fig. 3b). To further substantiate the data, we employed ultracentrifugation methodology to separate cytoplasmic, nuclear and membrane fractions from *N. benthamiana* leaves transiently expressing Desi3a-HA with either FLS2-GFP or GFP alone. Both FLS2 and Desi3a proteins were only present in the membrane fraction and were not detectable either in cytoplasmic or nuclear fractions (Supplementary Fig. 11).

Furthermore, we examined the in vivo interaction of Desi3a-HA with FLS2-GFP and FLS2^K/R^-GFP in the absence and presence of flg22 in *N. benthamiana*. As expected, Desi3a-HA coimmunoprecipitated with both FLS2-GFP and FLS2^K/R^-GFP but its interaction with FLS2 decreased considerably after flg22 treatment (Fig. 3c and Supplementary Fig. 22). However, no difference was observed in Desi3a interaction with the non-SUMOylatable FLS2^K/R^-GFP in the absence or presence of flg22. The reduced interaction with wild-type FLS2 upon flg22 treatment is, likely, due to changes in conformation that disrupts Desi3a-FLS2 interaction resulting in enhanced FLS2 SUMO conjugation, thereby serving as a positive feedback loop to trigger immune signalling. However, this Desi3a-FLS2 complex disruption did not happen with FLS2^K/R^-GFP as its no longer a substrate for SUMO conjugation.

To ascertain the specificity of Desi3a towards FLS2, we tested the sub-cellular localization and interaction of the Desi protease encoded at the loci *AT2G25190*, the closest homologue of Desi3a, with FLS2-GFP. The protein encoded by *AT2G25190* neither localized to cell membranes (but to the nucleus), nor showed any interaction with FLS2 (Supplementary Figure 12 and Supplementary Fig. 26) further supporting the notion that substrate specificity in the SUMO system is maintained by SUMO proteases.

Unlike ULPs, Desi SUMO proteases act only on the removal of SUMO conjugates, and not in SUMO maturation^35–37^. Therefore, we examined if Desi3a is capable of removing isopeptide-linked SUMO conjugates for it to be defined as a SUMO protease. We generated isopeptide-linked poly-SUMO chains^38^ and incubated them with GST-Desi3a as well as with GST-Desi3a^C168S^, which is Desi3a with the catalytic core Cysteine mutated to Serine. The in vitro deSUMOylation assay confirmed that Desi3a is, indeed, a bona fide Desi SUMO protease capable of cleaving isopeptide-linked SUMO chains with a catalytically important cysteine at position 168 (Fig. 3d). To ascertain if Desi3a can target FLS2 for deSUMOylation, we incubated GST-Desi3a and GST-Desi3a^C168S^ with polySUMOylated FLS2^KD^ (kinase domain where SUMO is conjugated). A reduction in higher molecular weight SUMO-conjugated isoforms of FLS2^KD^ was observed in the reaction that contained WT Desi3a protein and not in the reaction containing Desi3a^C168S^ protein (Supplementary Fig. 13).

Intrigued by the SUMO protease activity of Desi3a on FLS2, we speculated an involvement of Desi3a in immune signalling. To test this, we first compared the global SUMOylation levels between Col-0 and *desi3a-1* (SALK_151016C) at different time-points of flg22 treatment. *desi3a-1* was confirmed by comparing its transcript levels with that of Col-0 (Supplementary Fig. 14). We did not notice any significant difference in the SUMOylation levels of *desi3a-1*, as compared to Col-0 (Supplementary Fig. 15). This evidence suggested that rather than affecting globally, Desi3a is likely to have narrow range of targets, which may be difficult to detect in global SUMOylation immunoblots. Therefore, to analyse Desi3a specific role in immune response we compared transcript levels of key defence genes in Col-0 and *desi3a-1* upon flg22 and elf18 treatment. Interestingly, we observed a significant increase in the expression levels of the defence genes in *desi3a-1* compared to Col-0 for both flg22 and elf18 treatments (Supplementary Fig. 16a–f). This suggests an important role of Desi3a SUMO protease in regulating immune responses by repressing transcriptional responses downstream of plant PRRs.

Activation of defence genes in *desi3a-1* upon flg22 treatment prompted us to further investigate the impact of Desi3a deSUMOylation activity on FLS2-mediated immune signalling under in vivo conditions. Therefore, we generated transgenic plants expressing _*pro*_*FLS2::FLS2-GFP* in *desi3a-1* background and ascertained FLS2 SUMOylation levels. As expected, FLS2-GFP immunoprecipitated from *desi3a-1* background exhibited significantly higher levels of FLS2 SUMOylation as compared to FLS2-GFP complementing the *fls2* mutant background (Fig. 3e and Supplementary Fig. 22), further validating that Desi3a targets FLS2 for deSUMOylation. To examine if enhanced FLS2 SUMOylation in *desi3a-1* leads to increased BIK1 release and hence potentiated immune signalling, we compared ROS burst and MAPK activation in FLS2-GFP (in the *desi3a-1 mutant* background) transgenic lines with that of FLS2-GFP (complementing the *fls2* mutant) transgenic lines. Indeed, ROS burst levels (Fig. 3f) and MAPK activation (Fig. 3g and Supplementary Fig. 22) were significantly more in FLS2-GFP (*desi3a-1*) transgenic plants, offering strong support to the role of Desi3a in regulating FLS2-mediated immune responses by DeSUMOylation.

Intrigued by the enhanced SUMOylation of FLS2 in *desi3a-1*, we examined Desi3a protein levels in transgenic lines expressing *35S:Desi3a-HA* in *desi3a-1* mutant upon flagellin treatment. Interestingly, Desi3a-HA protein showed rapid degradation, within 10 min of flg22 treatment (Fig. 3h and Supplementary Fig. 22), highlighting a strong correlation between Desi3a turnover by flagellin and flagellin-induced FLS2 SUMOylation. As a direct proof of our findings that FLS2 SUMOylation is essential for BIK1 release from the immune complex and Desi3a negatively regulates this process by deSUMOylating FLS2, we used a gain-of-function approach by analysing FLS2-BIK1 interaction in the absence and presence of Desi3a. FLS2-GFP, BIK1-myc and SUMO1-HA were co-expressed either without or with Desi3a-HA in *N. benthamiana* in the presence of flg22. We observed, as expected, a clear release of BIK1 from the FLS2 complex in the presence of SUMO1 and flg22. On the contrary, we detect significantly reduced release of BIK1 when FLS2/BIK1/SUMO1 were co-expressed with Desi3a SUMO protease in the presence of flg22. This clearly demonstrates that BIK1 release from FLS2 relies on FLS2 SUMOylation. This evidence supports our claim that in the presence of Desi3a, which is a *bona fide* SUMO protease, FLS2 was deSUMOylated resulting in impaired dissociation of BIK1 from the immune complex (Supplementary Fig. 17).

The role of Desi3a in flagellin sensing was further supported by the observation that *desi3a-1* root growth was more sensitive to flg22; however, the growth inhibition phenotype was reverted in Desi3a-HA transgenic lines (Supplementary Fig. 18a, b).

### Desi3a regulates FLS2-mediated immune signalling

**Fig. 4:**
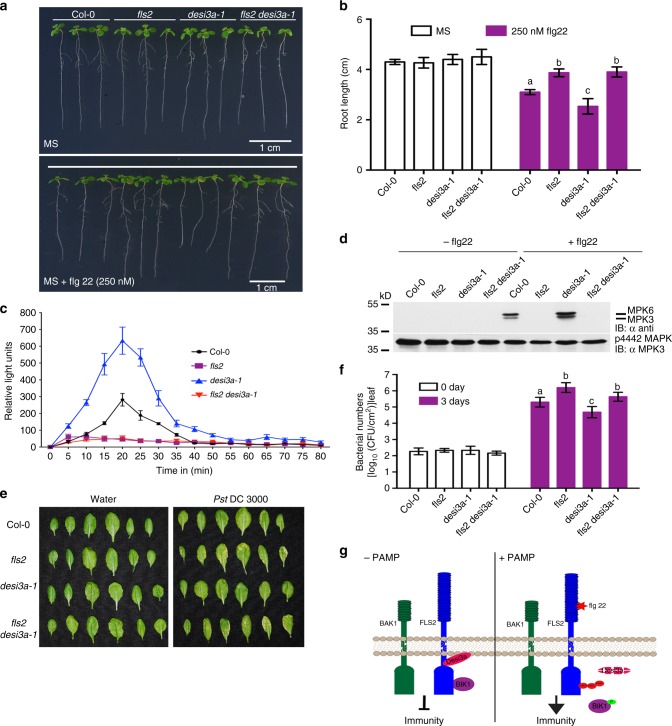
Desi3a functions downstream of FLS2 to regulate FLS2-mediated immune responses. **a** *fls2 desi3a-1* seedlings revert *desi3a-1* response to flagellin. A representative image of Col-0, *fls2, desi3a-1* and *fls2 desi3a-1* seedlings in 1/2MS or 1/2MS with 250 nM flg22. Scale Bar = 1 cm. **b** Quantification of root growth inhibition for Col-0, *fls2*, *desi3a-1* and *fls2 desi3a-1*. Four-day-old seedlings of the four genotypes were transferred to 1/2MS or 1/2MS + 250 nM flg22 and grown for additional 6 days and root lengths were measured. Data presented are means ± SE from at least 25 individual seedlings per genotype (Bars with different letters were significantly different from others; *P* < 0.05; two-way ANOVA with post hoc Tukey test). **c** *fls2 desi3a-1* shows reduced ROS production than *desi3a-1*. Leaf discs from three-week-old plants of Col-0, *fls2, desi3a-1* and *fls2 desi3a-1* were treated with 1 µM flg22 (10 min) and ROS burst was detected. Results shown are average ± SE (*n* = 3). **d** Enhanced MAPK activation in *desi3a-1* reverts to *fls2* levels in *fls2 desi3a-1*. Different genotypes were treated with water or 1 µM flg22 (10 min) and total proteins extracted were immunoblotted with anti-p44/42 MAPK antibody (upper panel) to detect activated MPK3/6, and with anti-MPK3 antibody for equal protein loading (lower panel). **e** *fls2 desi3a-1* shows enhanced sensitivity to bacterial pathogen *Pst* DC3000 than *desi3a-1*. Four-week-old rosettes of Col-0, *fls2*, *desi3a-1* and *fls2 desi3a-1* plants were dipped in liquid cultures of *Pst*. and leaves from rosettes infected with *Pst*. were photographed 5 days post-infection (dpi). **f** Quantification of colony-forming units (CFUs) from infected rosettes was counted at day 0 and 3 dpi. Values are means ± SE from three independent biological replicates (*N* = 3; bars with different letters were significantly different from others; *P* < 0.05; two-way ANOVA with post hoc Tukey test). **g** A schematic of flg22-mediated SUMO conjugation of the FLS2 receptor during PAMP signalling. In the presence of flg22 (+PAMP) FLS2 SUMOylation facilitates the release of BIK1 from the FLS2-BAK1 immune complex. This is concomitant with the flg22-dependant degradation of Desi type SUMO protease, Desi3a

The finding that Desi3a is crucial for FLS2 deSUMOylation prompted us to study the genetic interaction between the two genes. We genetically crossed the mutants *desi3a-1* and *fls2* to generate *fls2 desi3a-1* double mutant (Supplementary Fig. 19). To assess the genetic epistasis between Desi3a and FLS2 upon flagellin perception, we analysed flg22-mediated root growth inhibition of *fls2 desi3a-1* double mutant, and compared it to Col-0, *fls2* and *desi3a-1* single mutants in the presence of 250 μM flg22 (Fig. 4a, b). The *fls2 desi3a-1* double mutant showed root lengths comparable to *fls2*, thereby suppressing the flagellin sensitivity exhibited by *desi3a-1* (Supplementary Fig. 18). This clearly demonstrated that *fls2* is epistatic to *desi3a-1*. In addition, we compared the flg22-mediated ROS burst in the double mutant with Col-0 and the two single mutants. The ROS accumulation in *fls2 desi3a-1* double mutant was negligible, similar to *fls2*, whereas, it was highest for *desi3a-1* (Fig. 4c). Similarly, hyperactivation of the MAPK cascade after flg22 treatment was observed in *desi3a-1* compared to wild-type Col-0 plants, but no such activation was noticed in *fls2* or *fls2 desi3a-1* (Fig. 4d and Supplementary Fig. 23).

Adult plants of *desi3a-1* or *fls2 desi3a-1* double mutant showed no obvious morphological difference compared to Col-0 plants. However, pathogen infection assays with *Pst*. indicated that *desi3a-1* mutants were more resistant to virulent *Pst*. bacteria in comparison to Col-0. On the contrary, *fls2 desi3a-1* was more susceptible than both Col-0 and *desi3a-1* akin to fls2 mutants (Fig. 4e, f). Together this data indicated that flg22-mediated responses were potentiated in *desi3a-1* mutant and that Desi3a represents a negative regulator of FLS2-mediated immune signalling.

## Discussion

Compared to animals, plants have evolved an expanded number of receptor-like kinases (RLKs) with more than 600 members in Arabidopsis^39^. These kinases have been involved in sensing extrinsic and intrinsic signals to regulate plant growth and development^40^ along with their responses to the environment^5,19^. In general, these kinases contain a unique extracellular domain, transmembrane domain and cytoplasmic kinase domain^7^. Previously, it has been known that post-translational modification such as phosphorylation of cytoplasmic kinase domain transduces the signal to intracellular signalling networks^5,19,41^. Our results identify a SUMOylation site in kinase domain of FLS2. FLS2 SUMOylation neither affects the kinase activity of FLS2 nor its phosphorylation. Nonetheless, it is crucial for activating the FLS2-mediated immune signalling cascade. In silico analysis (Supplementary Fig. 1) showed no clear similarities in respect of SUMO site with other plant RLKs such as BAK1 and BIK1, which suggest that SUMOylation of RLKs is quite divergent and may have different roles and significance during plant defence.

Very few targets for these Desi type SUMO proteases have been identified to date. Indeed none of these SUMO proteases have been implicated in immunity across plant or animal kingdoms. Here, we demonstrate that in *desi3a-1* there was an enhanced SUMOylation of FLS2. Intriguingly flg22 treatment triggered a rapid reduction of Desi3a protein indicating that flg22 stimulates the accumulation of SUMOylated FLS2 by turnover of the negative regulator Desi3a. Taken together with the immunoprecipitation data we identify FLS2 as a target for Desi3a SUMO protease. The degradation of Desi3a upon flg22 treatment results in hyperSUMOylation of FLS2, triggering the timely release of BIK1 from the FLS2-BAK1 PRR complex to activate immune signalling in plants (Fig. 4g).

SUMOylation is an emerging PTM that affects a wide array of biological processes ranging from development to environmental sensing. Receptor kinases play critical roles in these processes. Presence of potential SUMO sites in these kinases suggests that SUMOylation of receptor kinases may act as a general mechanism for transducing activated transmembrane bound receptor complexes to transduce intracellular signalling events that regulate diverse biological processes.

## Methods

### Plant materials and growth conditions

*Arabidopsis thaliana* ecotype Columbia (Col-0) was used as the wild-type control plants. Arabidopsis plants used in this study were grown in environmentally controlled growth chambers as one plant per pot at 20–21 °C with a 10 h photoperiod for infection and ROS experiments and a 16 h photoperiod for generating and progressing transgenics. For growing on plates, Murashige and Skoog (MS) medium (Duchefa) with 1% agar was used and plants were grown at 22 °C with a 16 h photoperiod. The T-DNA SALK lines were obtained from the Nottingham Arabidopsis Stock Centre. The mutant lines used in this study are: *fls2* (SAIL_691C4) *desi3a-1* (SALK_151016C).

### Plasmid construction and plant transformation

All the constructs were generated by GATEWAY Cloning Technology. To generate the *FLS2*-GFP, *BAK1*-myc, *BIK1*-myc, *SCE1-HA*, *Desi3a-HA* (At1g47740.1) and *Desi3a-mCherry* constructs, the corresponding cDNA fragments were PCR-amplified and cloned into pENTR D-TOPO vector. By recombination all genes were moved to their final vector indicated in Supplementary Table 2. For generating _*pro*_*FLS2::FLS2-GFP*, 912 base pairs of FLS2 promoter, upstream of ATG was amplified using promoter specific primers and fused to full length CDS of *FLS2* gene (Supplementary Table 3).

To generate transgenic plants *fls2* plants were transformed with either _*pro*_*FLS2::FLS2-GFP* or _*pro*_*FLS2::FLS2*^*K/R*^*-GFP* constructs using *Agrobacterium*-mediated floral dip method. Similarly, *desi3a-1* was dipped with either _*pro*_*FLS2::FLS2-GFP* or 35 S:Desi3a-HA. To generate *fls2 desi3a-1* double mutant, single mutant *fls2* was crossed with *desi3a-1* single mutant to generate *fls2 desi3a-1* double mutant. The primers used to genotype the population are listed in Supplementary Table 3.

### Site-directed mutagenesis

Mutated versions of FLS2 was generated by site-directed mutagenesis using the pENTR/D-TOPO clones as template. Oligonucleotide primers used to introduce the mutations are listed in Supplementary Table 3. The introduction of mutations was confirmed by sequencing, performed both before and after introduction of the mutated FLS2 coding sequences into pMDC109 destination vector using LR Clonase (Invitrogen).

### Elicitors

Flg22 and elf18 peptides were purchased from Peptron (Peptron Inc, South Korea) and solubilized in sterile water to make stock solutions and diluted subsequently for the working concentrations.

### Measurement of reactive oxygen-species generation

Oxidative-burst measurement was performed^25^ by eliciting ROS with 1 µM flg22. Twelve leaf discs from three-week-old plants were used for each treatment, which was done by mixing leaf discs with buffer containing luminol (34 g/ml), horseradish peroxidase (20 g/ml) and flg22. Luminescence was measured over time using an ICCD photon-counting camera (Photek). The experiments were repeated at least three times.

### MAP kinase activation

MAPK assays were performed on 10-day-old seedlings grown in MS agar medium. Seedlings were elicited with 1 M flg22 or water for 10 min and frozen in liquid nitrogen. MAPK activation was monitored by western blot with antibodies that recognize the dual phosphorylation of the activation loop of MAPK (pTEpY). Phospho-p44/42 MAPK (Erk1/2) (Thr202/Tyr204) rabbit monoclonal antibodies from Cell Signaling (Cat. No. #4370; dilution: 1:2000) were used according to the manufacturer’s protocol. Total proteins were probed with anti-MPK3 antibody produced in rabbit (Merck, Cat. No. #M8318, dilution: 1:1000) to verify equal loading. The experiments were repeated at least three times.

### Seedling growth inhibition assay

Four-day-old seedlings of Col-0, *fls2*, *pFLS2:FLS2-GFP* and *pFLS2:FLS2*^*K/R*^*-GFP* and also of *desi3a-1, 35S:Desi3a-HA* and *fls2 desi3a-1* were transferred to MS plates with or without 250 nM flg22 and grown for additional 6 days before root length inhibition was measured. Col-0, *fls2*, seedlings were used as controls and two independent lines of each genotype were used in the seedling growth assays. At least 25 seedlings of each genotype per treatment were used for the experiment.

### Bacterial growth assays

Bacterial strain used in this study was *Pst* DC3000. *Pst* DC3000 was restreaked from a frozen glycerol stocks on to a Kings B Agar media (20.0 g Bacto Peptone, 0.75 g K_2_HPO_4_*7H_2_O, 10 ml glycerol L^−1^) plate with Kanamycin and Rifampicin antibiotics (50 mg L^−1^) and grown in dark for 2 days at 28 °C. For inoculation, *Pst* DC3000 was grown in Kings B broth at 28 °C overnight. Bacterial suspension was adjusted to 5 × 10^5^ colony-forming units (CFU)/ml in sterile MgCl_2_ (10 mM) containing 0.03% of the surfactant Silwet L-77^9^. Pathogen inoculation was performed by dipping 4-week-old plants into the bacterial suspension. The disease rate was scored at 72 h post-infection by determining the dark brown spots on the leaf surface and by counting the CFUs on Kings B medium plates. At least three samples for colony counting and 20 samples for disease rate scoring were taken for each treatment over a 3-day period.

### Total RNA extraction and quantitative RT-PCR

Twelve-day-old seedlings of different genotypes were treated with flg22 (1 µM) or elf18 (1 µM) along with mock (MS) and ground to a fine powder with mortar and pestle in liquid nitrogen. Spectrum^TM^ Plant Total RNA kit (Sigma-Aldrich) was used to extract RNA following the manufacturer’s recommendations. The RNA was quantified using NanoDrop^TM^ 1000 Spectrophotometer (Thermo Scientific) and about one microgram of total RNA was used for cDNA synthesis after DNase treatment with Promega DNase I. cDNA synthesis was undertaken with Invitrogen SuperScript-II Reverse Transcriptase following manufacturer’s guidelines.

Quantitative real-time PCR was conducted using Brilliant III Ultra-Fast SYBR QPCR master mix (Agilent) in conjunction with Rotor-Gene Q (Qiagen) and analysis was undertaken with the software provided using comparative quantification methods. *ACTIN7* (*At5g09810*) was used as the house-keeping gene for normalization. The experiments were repeated three times.

### Nuclear and membrane protein extraction

To prepare cytoplasmic, nuclear and membrane protein fractions^42^ plant samples were ground to a fine powder and homogenized in lysis buffer (20 mM Tris-HCl, pH 7.4, 25% glycerol, 20 mM KCl, 2 mM EDTA, 2.5 mM MgCl_2_, 250 mM Sucrose). The homogenate was sequentially filtered through 100 and 30 µm nylon mesh. The fraction was collected for further analysis for cytosolic protein analysis using anti-UGPase rabbit polyclonal antibody (Agrisera, Cat. No. AS05086, dilution: 1:2000). The nuclei were pelleted by centrifugation at 1500 × *g* for 10 min and washed three times with nuclei suspension buffer (20 mM Tris-HCl, pH 7.4, 25% glycerol, 2.5 mM MgCl_2_, 0.2% Triton X-100). The nuclear fraction collected and used to analyse the nuclear protein using anti-histone H3 rabbit polyclonal antibody (Abcam, Cat. No. ab1791, dilution: 1:3000). The cytosolic fraction was centrifuged at 16000 × *g* and the collected supernatant was subjected to ultracentrifugation (100,000 × *g* for 1 h) and the pellet was used to analyse the membrane protein.

### Immunoprecipitation and coimmunoprecipitation assay

Arabidopsis plants were dipped in 10 mM MgCl_2_ or 1 μM flg22 for 10 min and total protein was isolated for IP using the extraction buffer containing 100 mM Tris-HCl, pH 8.0, 0.1% [w/v] SDS, 0.5% [w/v] sodium deoxycholate, 1% [v/v] glycerol, 50 mM sodium metabisulfite, 20 mM N-ethylmaleimide (NEM) and protease inhibitor cocktail (Roche). Anti-GFP IP was performed. Total protein was incubated with 50 μl anti-GFP beads (Chromotek anti-GFP beads) and incubated on ice for 30 min. The beads were centrifuged down at 10,000 × *g* for 1 min and washed three times with 1 ml of cold IP buffer. After the last wash 50 μl of pre-heated (95 °C) 1 × SDS-loading buffer was used to elute the immuno-complex and analysed on 10% SDS-PAGE using immunoblotting methods with rabbit polyclonal anti-GFP antibody (Abcam, Cat. No. ab6556, dilution: 1:6000) and anti-SUMO1/2 antibodies generated against AtSUMO1 in rabbit. The experiments were repeated at least three times.

*N. benthamiana* plants were infiltrated with 10 mM MgCl_2_ or 5 μM flg22 for 15 min, total protein was isolated for co-IP using the extraction buffer containing 50 mM HEPES (pH 7.5), 1 mM EDTA, 0.5% Trition X-100, 1 mM DTT. Anti-GFP IP and anti-myc IP were performed. Total protein was incubated with 50 μl anti-GFP beads (Chromotek anti-GFP beads) and incubated on ice for 30 min. The beads were centrifuged down at 10,000 × *g* for 1 min and washed three times with 1 ml of cold IP buffer. After the last wash 50 μl of pre-heated (95 °C) 1 × SDS-loading buffer was used to elute the immuno-complex and analysed on 10% SDS-PAGE using immunoblotting methods with anti-GFP (Abcam), anti-HA rat monoclonal antibody (Roche, Cat. No. 11867423001, dilution: 1:5000) and anti-myc mouse monoclonal antibody (Invitrogen, Cat. No. 13-2500, dilution: 1:3000). The experiments were repeated three times.

### Proteomic screen

We generated transgenic Arabidopsis lines expressing 35S::Strep-SUMO1^H89R^ in Col-0 background, since the H89R SUMO1 variant simplified MS/MS detection of SUMO-conjugated lysines after trypsinization^43^. The plasmid details are included in Supplementary Table 2. Transgenic seedlings were treated with water or 250 nM flg22 for 30 min and total protein extracts including membrane fractions were affinity purified with Pierce™ Streptavidin Magnetic Beads. Transgenic Col-0 plants expressing 35S::StrepII tag alone were used as negative controls for cross reacting streptavidin binding proteins. Total protein was extracted from five grams of 14-day-old seedling tissue from transgenic plants using buffer conditions as indicated above and subjected to affinity purification with magnetic beads as per manufacturer’s protocol. After a short wash step, elution of purified proteins was performed by addition of a low-pH glycine buffer (0.1 M glycine, pH 2.0). The resulting elutes were trypsin digested, desalted and concentrated using Ekspert nano LC 400 (EKSIGENT). The peptides were subsequently subjected to TOF-TOF mass spectrometry (SCIEX 6600) analysis. Peptide sequences were assigned using ProteinPilot software (SCIEX) against the *Arabidopsis* protein database. The identified peptides were cross-matched to those derived from strep-tag alone controls to rule out non-specific targets. The experiment was repeated twice for confirmation. The mass spectrometry proteomics data have been deposited to the ProteomeXchange Consortium via the PRIDE partner repository with the dataset identifier PXD011552.

### SUMO chain formation and deSUMOylation assay

Isopeptide-linked poly-SUMO1 chains were constructed using the *Arabidopsis thaliana* SUMO machinery components. His-SAE1 (SUMO E1), MBP-PIAL2 (SUMO E4) and His-SUMO1 were purified using His and MBP affinity Trap columns (GE Healthcare), whereas SCE (SUMO E2) was purified by ion exchange chromatography using a HiTrap column (GE Healthcare). After purification all components underwent buffer exchange into SUMO buffer (150 mM NaCl, 20 mM Tris pH 7.5, 5 mM ATP, 5 mM MgCl_2_), and quantified. For SUMO chain formation, 4 μg SAE, 1 μg SCE, 100 μg SUMO proteins, 5 μg PIAL2 and 5 mM ATP (pH 7.5) were incubated in SUMO buffer at 30 °C for 4 h. Subsequently, the poly-SUMO1 chains were purified using His-Bind resin (Novogen) and used for deSUMOylation assays.

The deSUMOylation reaction was carried out as indicated in Tomanov et al.^38^, with minor modifications. About 10 μM purified His-SUMO isoforms or SUMOylated GST-FLS2^KD^ was incubated with 5 μM of the GST-Desi3a or GST-Desi3a^C168S^ in deSUMOylation buffer (150 mM NaCl, 50 mM Tris pH 8.0, 1 mM DTT and 0.2% Igepal) at 30 °C for 16 h. The reactions were stopped by adding 4× SDS sample buffer and heating to 98 °C for 3 min. Samples were subjected to 10% SDS_PAGE and immunoblotted with anti-SUMO1/2 (rabbit) and anti-GST rat monoclonal antibody (Merck, Cat. No. SAB4200055, dilution: 1:5000).

### Protein extraction and western blot analysis

Frozen plant tissue was ground to a fine powder with a chilled pestle and mortar. Protein extraction buffer (50 mM Tris/HCl, pH 8.5, 4% SDS, 2% β-mercaptoethanol, 10 mM EDTA) and protease inhibitor tablet was added 1:1 w/vol. The mixture was centrifuged at 12,000 × *g* at 4 °C for 10 min. The protein concentration was determined using a Direct Detect TM Infra-red Spectrometer (EMD Millipore) and samples were equalized with the addition of extraction buffer. Protein loading dye (4 ×) was added and the samples were separated on polyacrylamide gels. The proteins were transferred to a polyvinylidene difluoride (PVDF) membrane and blocked with 5% semi-skimmed milk powder at room temperature and probed with the respective antibodies. Secondary horseradish peroxidase (HRP)-conjugated antibodies were applied before developing the blots with X-ray film using an automated developer. The blots used in the study are present in the Supplementary Info after the tables.

### Confocal microscopy analysis and imaging

For transient expression, four-week-old *N. benthamiana* plants were infiltrated on the abaxial side of the leaf with infiltration media (10 mM MgCl_2_ and 150 μg/ml acetosyringone) and *Agrobacterium tumefaciens* bacteria suspended in infiltration media. Agrobacterium cultures were prepared following a published protocol^44^. Agrobacterium harbouring expression constructs were infiltrated at an OD600 of 0.1 into *N. benthamiana* 72 h prior to confocal imaging. Sections of *N. benthamiana* leaves transiently expressing FLS2-GFP, FLS^K/R^-GFP or GFP only and/or mCherry-Desi3a proteins were randomly sampled and mounted in perfluoroperhydrophenanthrene (PP11). Imaging was conducted with Zeiss LSM 880 laser scanning confocal microscope (LSCM) with Airyscan module. The excitation wavelength was 488 nm for GFP, and 594 nm for mCherry. Emission was detected using BP 495–550 nm for GFP and LP 605 nm filter for mCherry, airyscan processing was done using automatic Weiner filter settings.

### Statistical analysis

All statistical analysis was performed using GraphPad Prism 6 software. One-way or two-way ANOVAs with post hoc Tukey test were performed at a significance level of *P* < 0.05 or *P* < 0.01 or *P* < 0.001. All root phenotype experiments had at least an *n* = 25–30 seedlings in each biological replication. Data are representing an average of three individual biological replicates.

## Electronic supplementary material

Reporting Summary

Supplementary Data

Source Data

## Data Availability

The mass spectrometry proteomics data associated with this study have been deposited to the ProteomeXchange Consortium via the PRIDE partner repository with the dataset identifier PXD011552. The uncropped versions of blots shown in the study are provided in the Supplementary Figures 20–26. Source Data pertaining to Fig. 1d, Fig. 1f, Fig. 2c, Fig. 3b, Fig. 4c, Fig. 4d, and Fig. 4f are provided in a Source Data file. The authors declare that all other data supporting the findings of this study are available within the manuscript and its supplementary files or are available from the corresponding author upon request.
