# Supplementary material for: SUMO conjugation to the pattern recognition receptor FLS2 triggers intracellular signalling in plant innate immunity
Source: Nat Commun. 2018 Dec 5;9:5185. doi: 10.1038/s41467-018-07696-8 (PMC6281677; doi:10.1038/s41467-018-07696-8)
Supplement: Supplementary file 2 — Supplementary Data [file 41467_2018_7696_MOESM2_ESM.pdf]

```
AtFLS2     HVSDFGTARILGFREDGSTTASTSAFEGTIGYLAPEFAYMRKVTTKADVFSFGIIMMELM  1071
AhFLS2     HVSDFGTARILGFREDGSTTASTAAFEGTIGYLAPEFAYMSKVTTKADVFSFGIIMMELM  1073
BrFLS2     HVSDFGTARILGLREDGSVTASTLAFEGTIGYLAPEFAYMRKVTTKADVFSFGIVMMELM  1073
BoFLS2     HVSDFGTARILGLREDGSVTASTLAFEGTIGYLAPEFAYMRKVTTKADVFSFGIVMMELM  1075
EsFLS2     HVSDFGTARILGLREDGSITASTSVFEGTIGYLAPEFAYMRKVTTKVDVFSFGIIMMELM  1077
AlFLS2     HVSDFGTARILGFREDGSTTASTAAFEGTIGYLAPEFAYMSKVTTKADVFSFGIIMMELM  1073
CrFLS2     HVSDFGTARILGLREDGSTTASTLAFQGTIGYLAPEFAYMRKVTTKADVFSFGIIMMELM  1071
CgFLS2     HVSDFGTARILGLREDGSTTASTLAFQGTIGYLAPEFAYMRKVTTKADVFSFGIIIMELM  1071
BsFLS2     HVSDFGTARILGLREDGSTTASTSAFEGTIGYLAPEFAYMRKVTTKADVFSFGIIMMELM  1073

AtFLS2     TKQRPTSLNDEDSQDMTLRQLVEKSIGNGRKGMVRVLDMELGDSIVSLKQEEAIEDFLKL  1131
AhFLS2     TRQRPTSLNDEKSQGMTLRQLVEKSIGDGTEGMIRALDSELGDAIVTRKQEEAIEDLLKL  1133
BrFLS2     TKRRPTSVIDEESQGVSLRQLVEEAVGDGVEGMIRVLDSEIGLSIVTRKQEEAVEDLLKL  1133
BoFLS2     TKRRPTSVIDEESQGVSLRQLVEEAIGDGVEGMIRVLDSEIGLSIVTRKQEEAVEDLLKL  1135
EsFLS2     TKQRPTSLSDEESQGVTLRQLVEKSIGEGTEGIIRVLDSEIRASIVSRKQEEAIEDLLKL  1137
AlFLS2     TRQRPTSLNDEKSQGMTLRQLVEKSIGDGTEGMIRVLDSELGDAIVTRKQEEAIEDLLKL  1133
CrFLS2     TKQRPTSLNEEKSQGMTLRQLVEKSIEDGTEGIIRVLDSELGDDIVTRKQEEAVEDLLKL  1131
CgFLS2     TKQRPTSLNEEKSQGMTLRQLVEKSIEDGTEGIIRVLDSELGDTIVTRKHEEAVEDLLKL  1131
BsFLS2     TKQRPTSLNDEESQGMTLRQLVEKSIGDGTEGMIRVLDSELGDAIVTRKQEEAIEDLLKL  1133
```

Supplementary Figure 1

Amino acid alignment of the kinase domain of FLS2 from *Arabidopsis thaliana* with *Arabidopsis halleri* (*Ah*FLS2), *Brassica rapa* (*Br*FLS2), *Brassica oleracea* (*Bo*FLS2), *Eutrema salsugineum* (*Es*FLS2), *Arabidopsis lyrata* (AlFLS2), *Capsella rubella* (*Cr*FLS2), *Capsella grandiflora* (*Cg*FLS2) and *Boechera stricta* (*Bs*FLS2) and The conserved lysine in the kinase domain is highlighted in grey. Phytozome (https://phytozome.jgi.doe.gov/pz/portal.html) was used to obtain the FLS2 homolog sequences for different plant species.

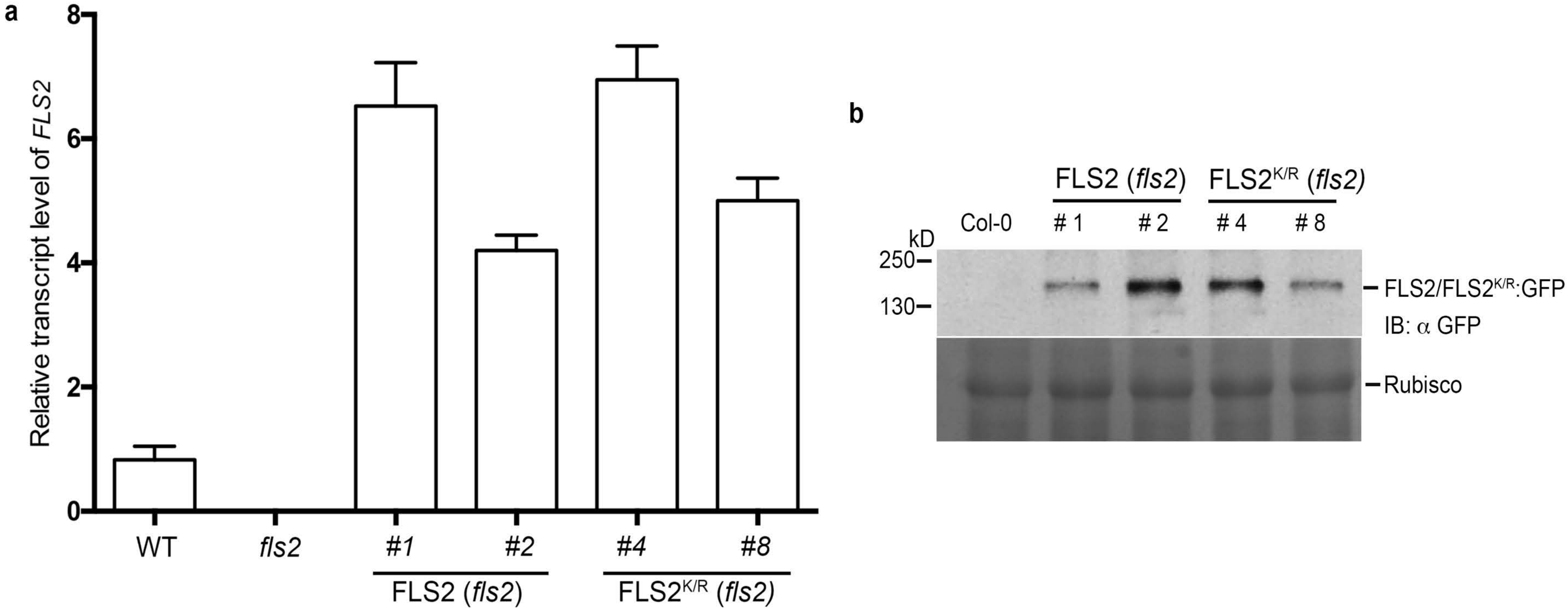

Supplementary Figure 2

Expression analysis of FLS2 in different transgenic lines (a) The expression levels of FLS2 in Col-0, *fls2* and different transgenic plants by real-time PCR analysis. The experiment was replicated three times and data shows mean ± SD (N = 3). (b) The protein level of FLS2-GFP and FLS2$^{K/R}$-GFP in different transgenic plants by western blot analysis. Blots were probed with anti-GFP antibody.

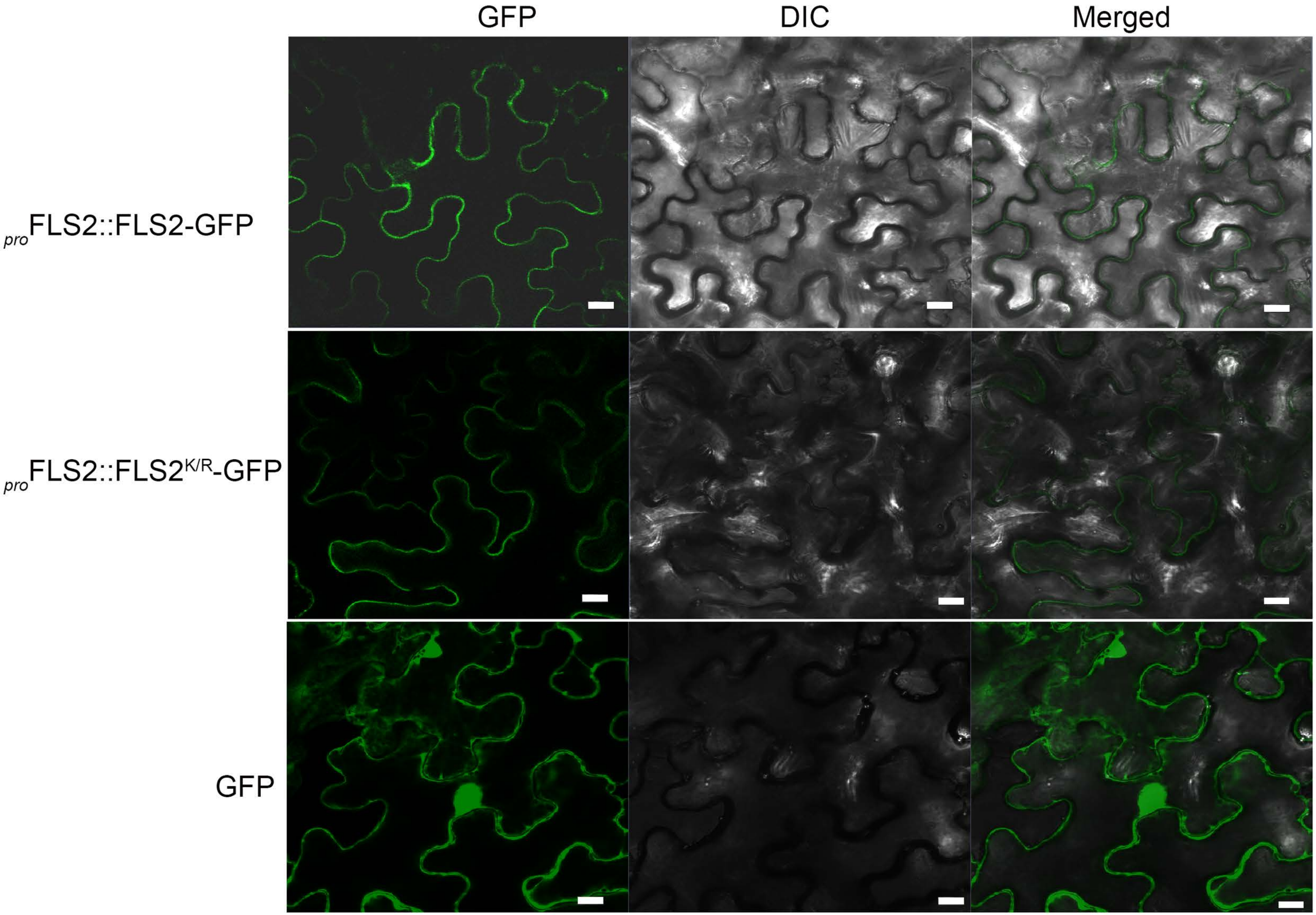

Supplementary Figure 3

Loss of the SUMO acceptor site K1120 does not affect FLS2 localization. Localization of FLS2-GFP and FLS2$^{K/R}$-GFP fusion proteins in *N. benthamiana* leaves was analyzed by confocal laser scanning microscopy. The localization pattern does not alter after mutation of lysine to arginine. The green signals indicate GFP and differential interference contrast (DIC) images were overlapped with GFP images (Merged). Scale bar = 10µm

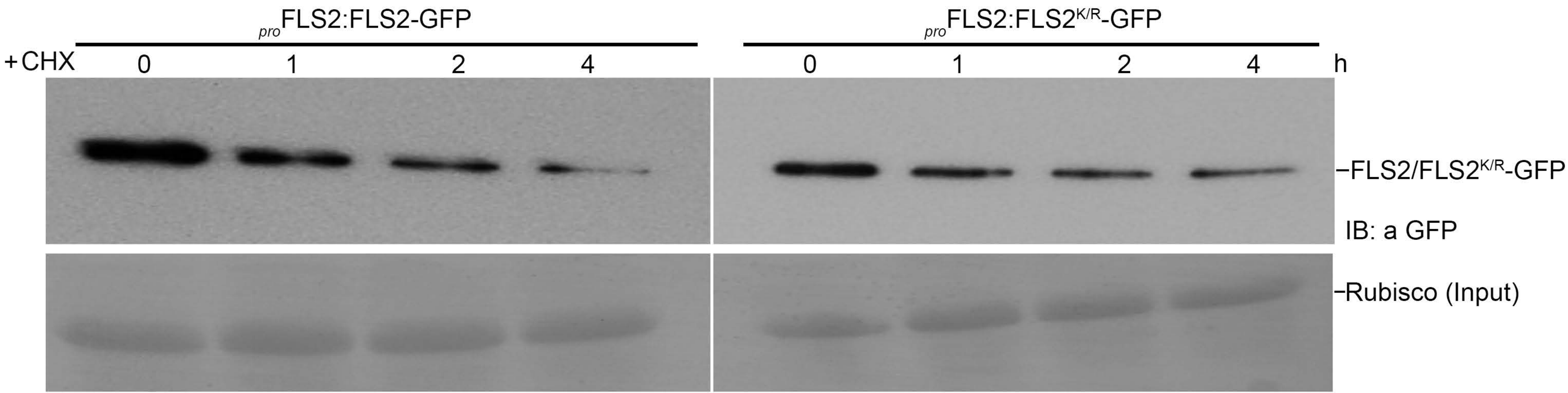

Supplementary Figure 4

FLS2 protein degradation is not affected due to mutating the conserved lysine residue that is conjugated to SUMO. Immnuoblot showing that FLS2-GFP (line #2) and FLS2$^{K/R}$-GFP (line #4) degradation is comparable. Proteins were extracted from 10-day-old cycloheximide (CHX) treated transgenic seedlings at different time points. Blot probed with anti-GFP antibody. Rubisco used as loading control.

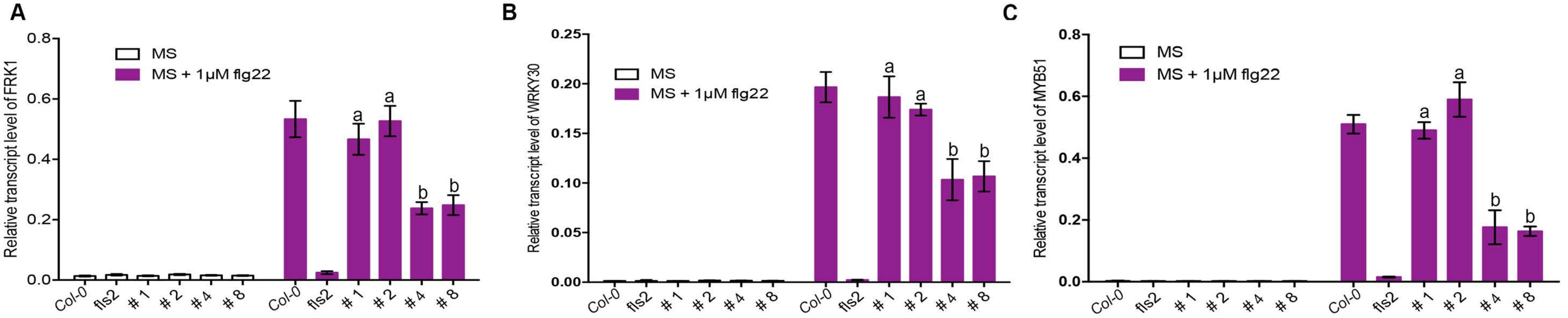

Supplementary Figure 5

Quantitative RT-PCR analyses of gene expression after flg22 treatment in Col-0, *fls2*, $_{pro}$FLS2:FLS2-GFP and proFLS2:FLS2$^{K/R}$-GFP seedlings. Total RNA was extracted from 10-day-old seedlings treated with 1μM of flg22 for 2 hours. The actin transcript was analyzed as internal control. Error bars are standard deviation of three biological replicates. Bars with different letters were significantly different from others (*P< 0.05; **P< 0.01).

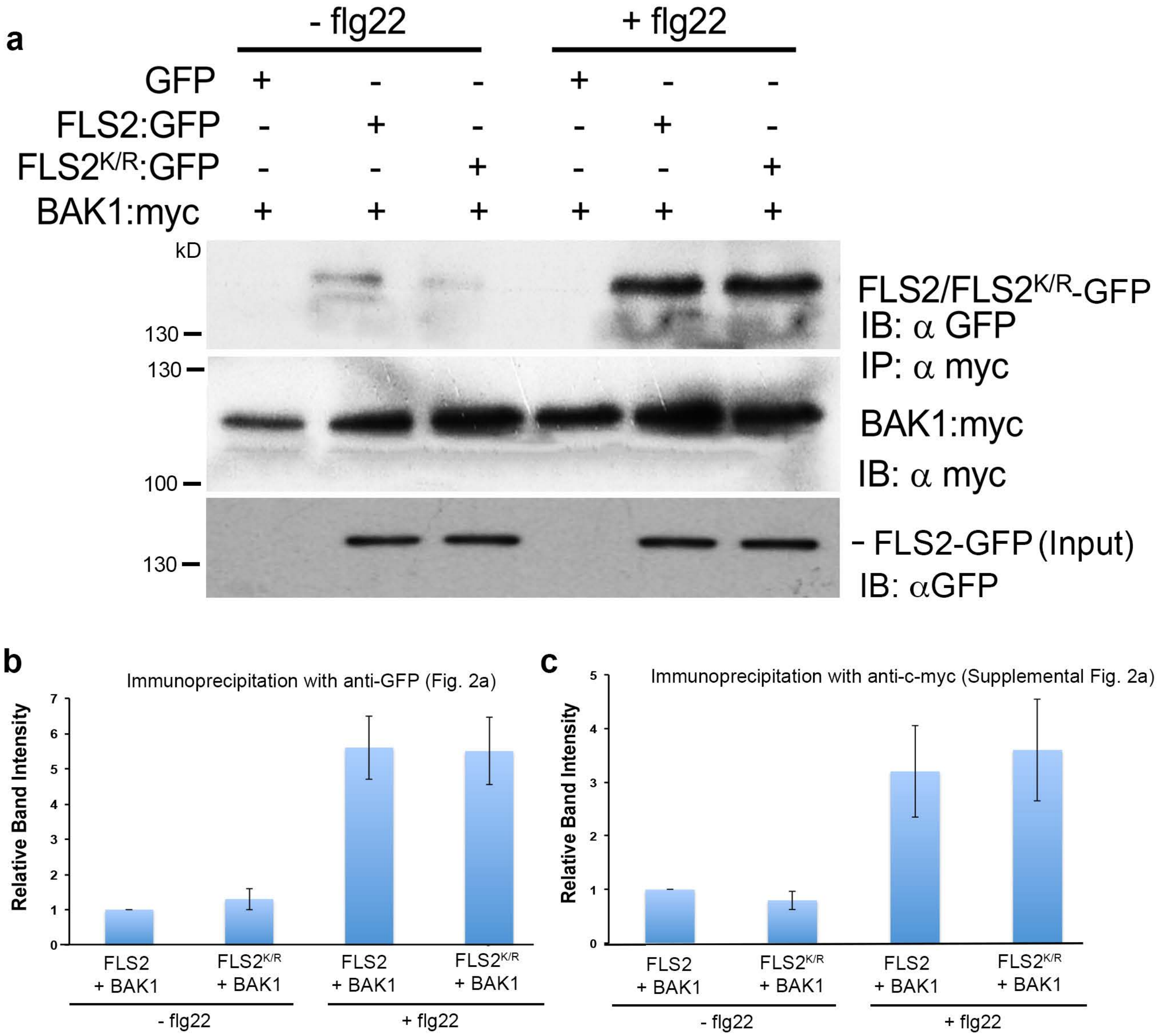

Supplementary Figure 6

FLS2 and FLS2$^{K/R}$ forms a complex with BAK1 in *N. benthamiana*. **(a)** Coimmunoprecipitation (Co-IP) analysis from transiently expressed proteins in *N. benthamiana* leaves showing flg22 dependent complex between FLS2-BAK1 and FLS2$^{K/R}$-BAK1. FLS2/ FLS2$^{K/R}$ is fused to GFP and BAK1 is fused to myc tag. Transiently expressing GFP alone was used as a negative control. Immunoblot showing interaction of BAK1 with both FLS2/ FLS2$^{K/R}$ in the presence of flg22. **(b)** Quantification of the immunoblots from two independent biological replicates of FLS2 BAK1 pull down assays where immunoprecipitation was done using anti-GFP antibody beads (a representative blot is shown in Fig. 2a). BAK1-myc pulled down with FLS2 WT in the absence of flg22 was taken as reference and denoted as 1. The BAK1-myc blots were normalized to respective FLS2-GFP bands. **(c)** Quantification of the immunoblots from two independent biological replicates of FLS2 BAK1 pull down assays where immunoprecipitation was done using anti-myc beads (a representative blot is shown in Supplementary Fig. 6a). FLS2-GFP pulled down with BAK1-myc WT in the absence of flg22 was taken as reference and denoted as 1. The FLS2-GFP blots were normalized to respective BAK1-myc bands. ImageJ software was used for all the band intensity measurements.

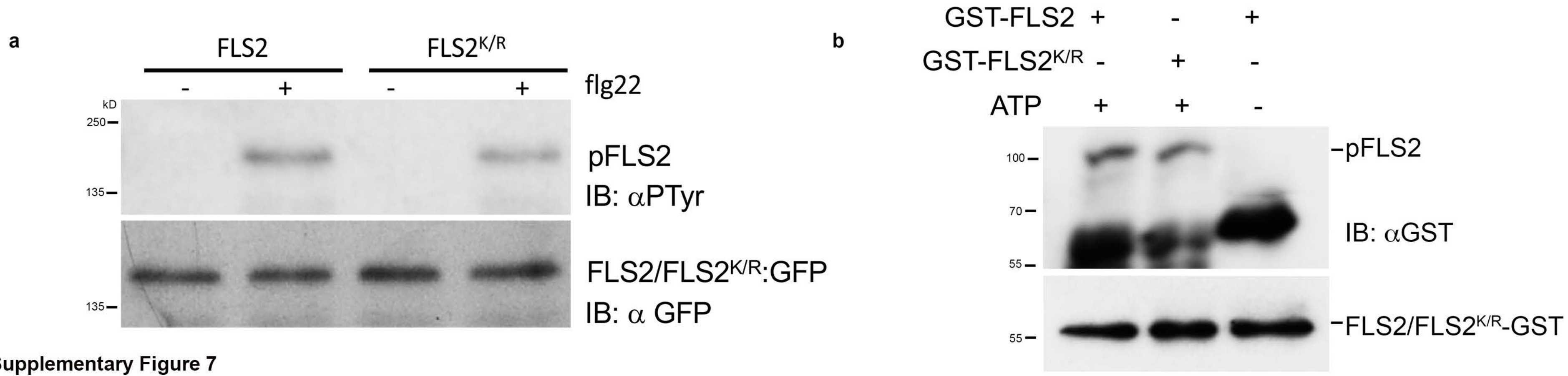

Supplementary Figure 7

FLS2$^{K/R}$-GFP is not affected in auto- or trans-phosphorylation abilities. **(a)** Trans-phosphorylation of FLS2 is not affected after SUMO site mutation. FLS2-GFP and FLS2$^{K/R}$-GFP proteins were immunoprecipitated from water- or flg22-treated transgenic seedlings and immunoblotted with anti-GFP to observe the fusion protein levels and anti-phosphotyrosine antibody was used to detect the phosphorylated proteins. **(b)** FLS2$^{K/R}$ auto-phosphorylation is similar to FLS2. Purified recombinant kinase domains of GST-FLS2 and GST-FLS2$^{K/R}$ proteins were incubated with ATP in the kinase buffer for 1 h at 25 °C. Samples were then subjected to Phos-tag gel analysis, which slows migration of phosphorylated proteins. Blots were probed with anti-GST antibody.

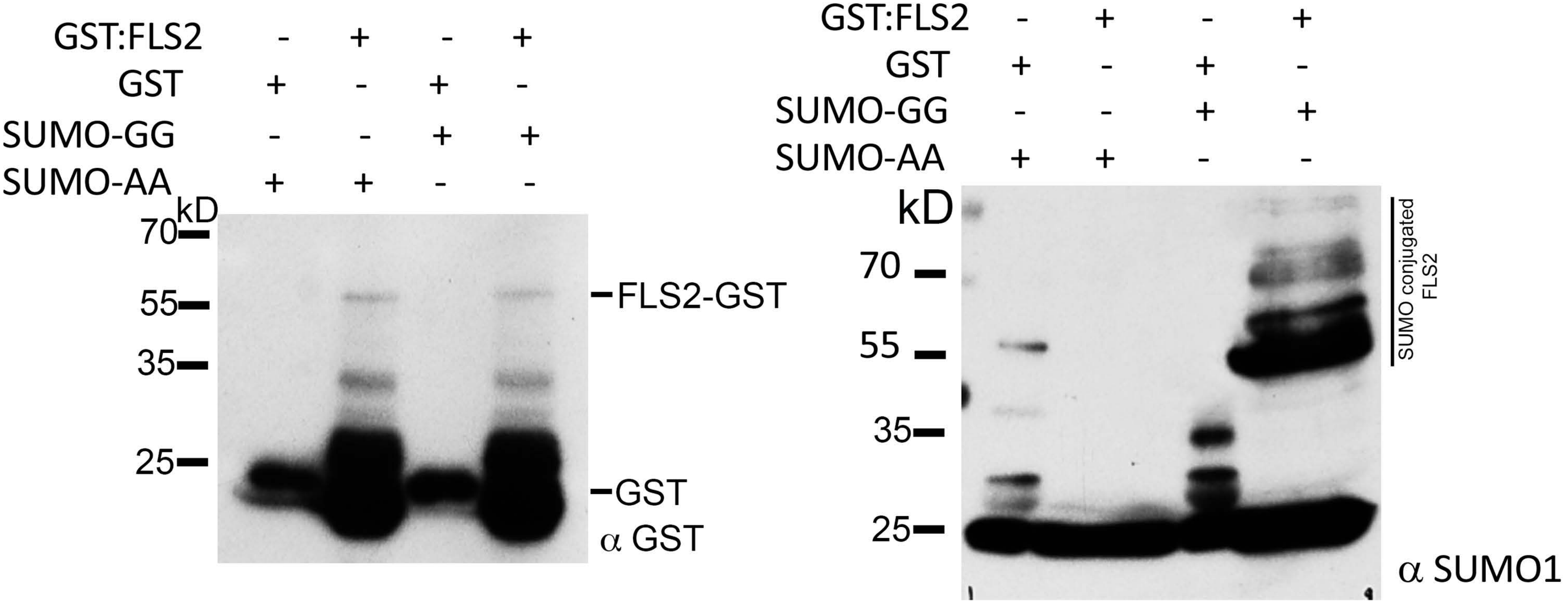

Supplementary Figure 8

In vitro SUMOylation assays using the reconstituted SUMOylation system in *E. coli* harbouring pCDFDuet-AtSUMO1 (GG)-AtSCE1a, pACYCDuet-AtSAE1a-*At*SAE2 and pDEST15-FLS2 were cultured in the presence of IPTG. Total lysates were incubated with GST-resin, and the proteins bound to the resin were analyzed by 10% SDS-PAGE. For negative controls, *E. coli* harboring pCDFDuet-AtSUMO1 (AA)-*At*SCE1a, pACYCDuet-AtSAE1a-AtSAE2 with pDEST15 or pDEST15-FLS2 were used. Blots were probed with anti GST and anti SUMO1 antibodies.

a

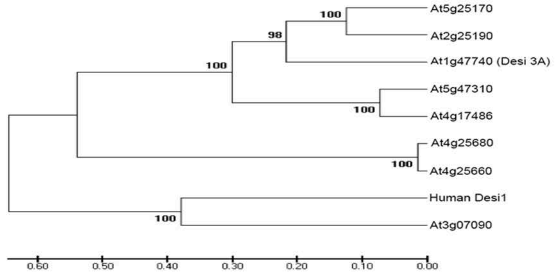

b

```
At3g07090            GVWHTGIVVYGN-EYFFG------GGIQHLPVGRTPYGTPIRTIELGLSHVPKDVFEMYLEEISP-RYTAESYNLLTHNCNNFSNEVAQ--  81
Human Desi1          GIWHTSIVVHKD-EFFFGS-----GGISSCPPGGTLLGPPDSVVDVGSTEVTEEIFLEYLSSLGESLFRGEAYNLFEHNCNTFSNEVAQ--  83
At4g25680            GIFHSAIQVYGNDEWSYGYCE-LGTGVFSCPSGKNPMYTYREKIVLGKTDCTIFMVNQMLRELSR-EWPGHTYDLLSKNCNHFCDVLCD--  87
At4g25660            GIFHSAIQVYGNDEWSYGYCE-QGTGVFSCPSGKNPMYTYREKIVLGKTDCTIFMVNQILRELSR-EWPGHTYDLLSKNCNHFCDVLCD--  87
At4g17486            GIFHSGIEAHNL-EYCYGAHEYPTSGVYEVEPRNCPGFIFRRSVLLGTTSMSRSDFRSYMEKLSR-KYHGDTYHLIAKNCNHFTEEVCL--  87
At5g47310            GIFHSGIEAHGF-EYGYGAHEYSSSGVFEVEPRSCPGFIFRRSVLLGTTSMSRSDFRSFMEKLSR-KYHGDTYHLIAKNCNHFTEEVCL--  87
At1g47740 (Desi 3A)  GIFHSGVEVHGV-EYAFGAHDYATSGVFEVEPRQCPGFKFKKSIFIGTTNLNPTQVREFMEDMAC-SYYGNMYHLIVKNCNHFCQDVC---  86
At5g25170            GIYHSGVEVHGV-EYGFGAHDHSTTGIFEVEPKQCPGFTFRKSILIGRTDLDPENVRVFMEKLAE-EYSGNSYHLITKNCNHFCNDVCVQL  89
At2g25190            GVFHSGVEVHGV-EYAFGAHESSSTGIFEVEPKKCPGFTFRKSILVGKTDLVAKEVRVFMEKLAE-EYQGNKYHLITRNCNHFCNEVCL--  87
                        *                                                                        *
```

Supplementary Figure 9

Identification of Arabidopsis Desi type SUMO protease. (a) Molecular phylogenetic analysis of the *Arabidopsis* Desi proteins was performed using the maximum likelihood method. Evolutionary history inferred based on the Jones et al. (w/freq. model). The human desi-1 protein was used as a positive control for the homology analysis. (b) Amino acid alignment of the catalytic domain of Desi type SUMO proteases indicating conserved active site NCN and H residues (asterisks).

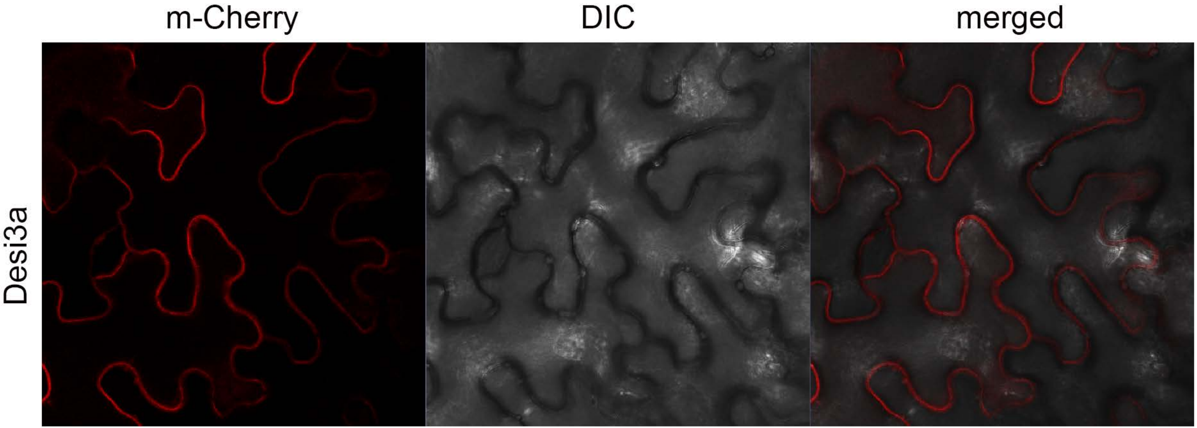

Supplementary Figure 10
Desi3a is localized to the plasma membrane. *Nicotiana benthamiana* leaves were co-infiltrated with mCherry-Desi3a. Fluorescent signals were detected after three days using confocal laser scanning microscope Carl Zeiss 880. Scale bar=10 μm.

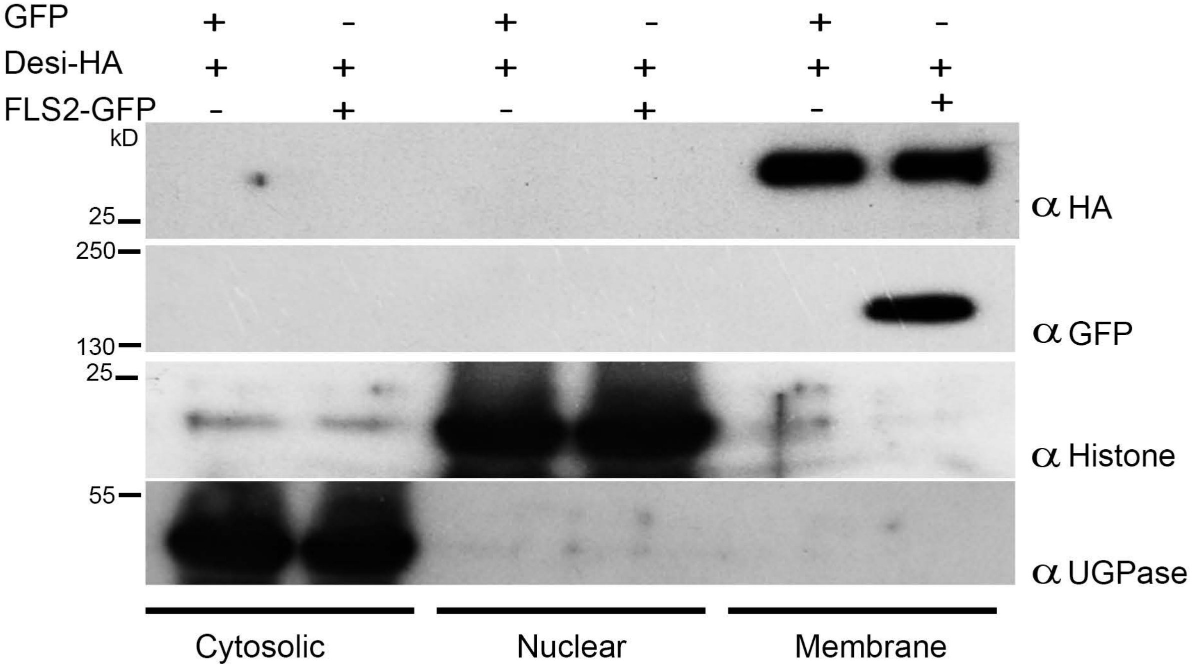

Supplementary Figure 11

FLS2 and Desi3a protein accumulates in the membrane. Cytosolic, nuclear and membrane proteins were extracted from transiently co-expressed FLS2-GFP and Desi3a-HA in *N. benthamiana* leaves. Protein extracts from different subcellular protein fractions was extracted using ultracentrifugation and analysed by SDS-PAGE followed by immunoblotting with anti GFP, anti HA, anti Histone (control for nuclear protein) and anti UGPAse (control for cytosolic protein) antibodies.

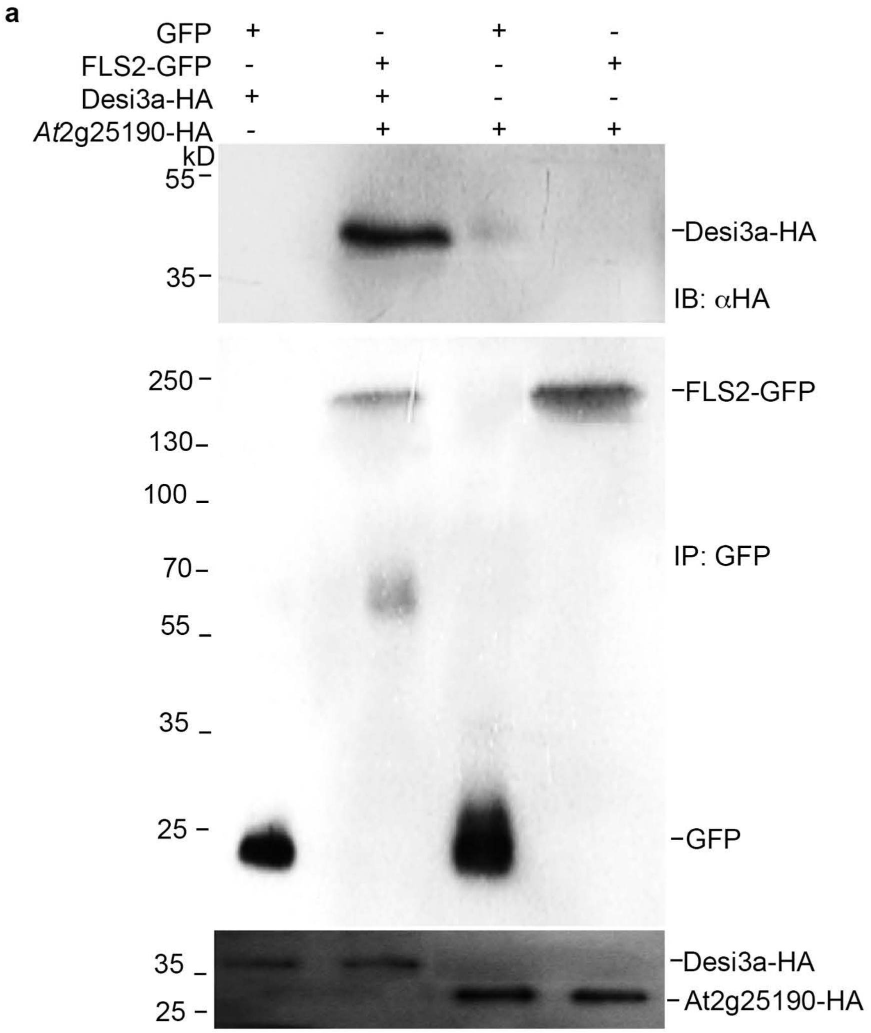

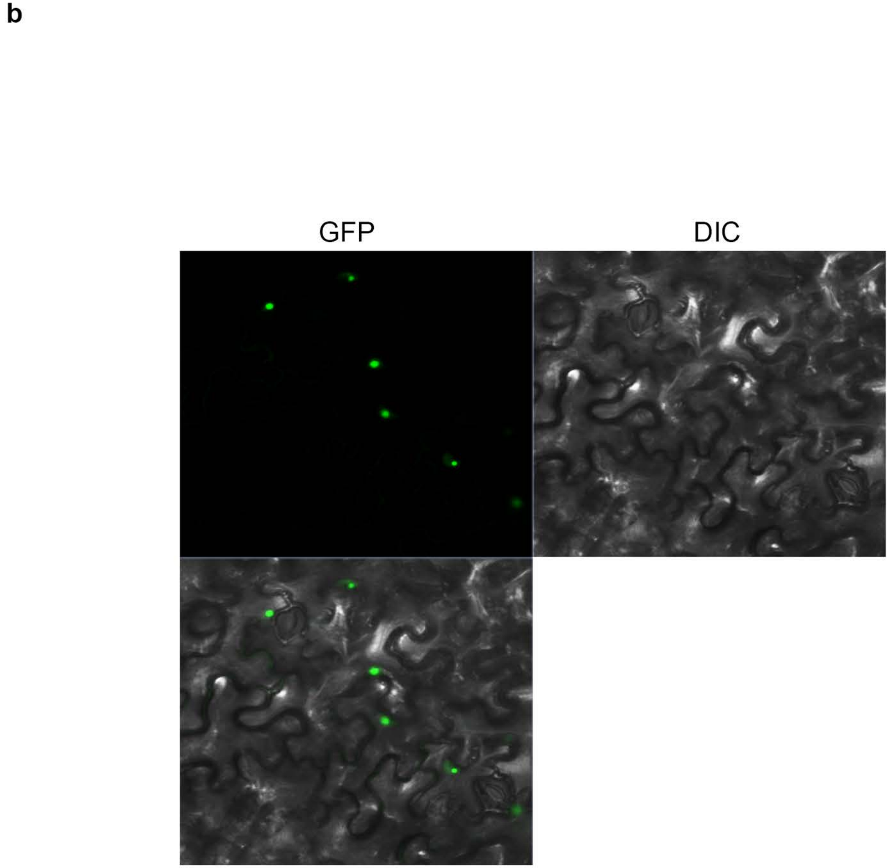

Supplementary Figure 12

FLS2 interacts with Desi3a but not other closest homologue. (A) Co-immunoprecipitation (co-IP) assays reveal that FLS2 interacts with Desi3a but not with At2g25190. Upon transient co-delivery of GFP, FLS2-GFP and Desi3a-HA and At2g25190-HA in *N. benthamiana* leaves. Samples were immunoprecipitated with anti-GFP beads. Specific proteins were detected by immunoblotting with the anti-GFP and anti-HA antibodies. All the experiemtns were repeated at least three times with similar results. (B) Localization of transiently expressed GFP-At2g25190 fusion proteins in *N. benthaiana* leaves was analyzed by confocal laser scanning microscopy. It localize in the nucleus. The green signals indicate GFP and differential interference contrast (DIC) images were overlapped with GFP images (Merge). Scale bar = 10μm

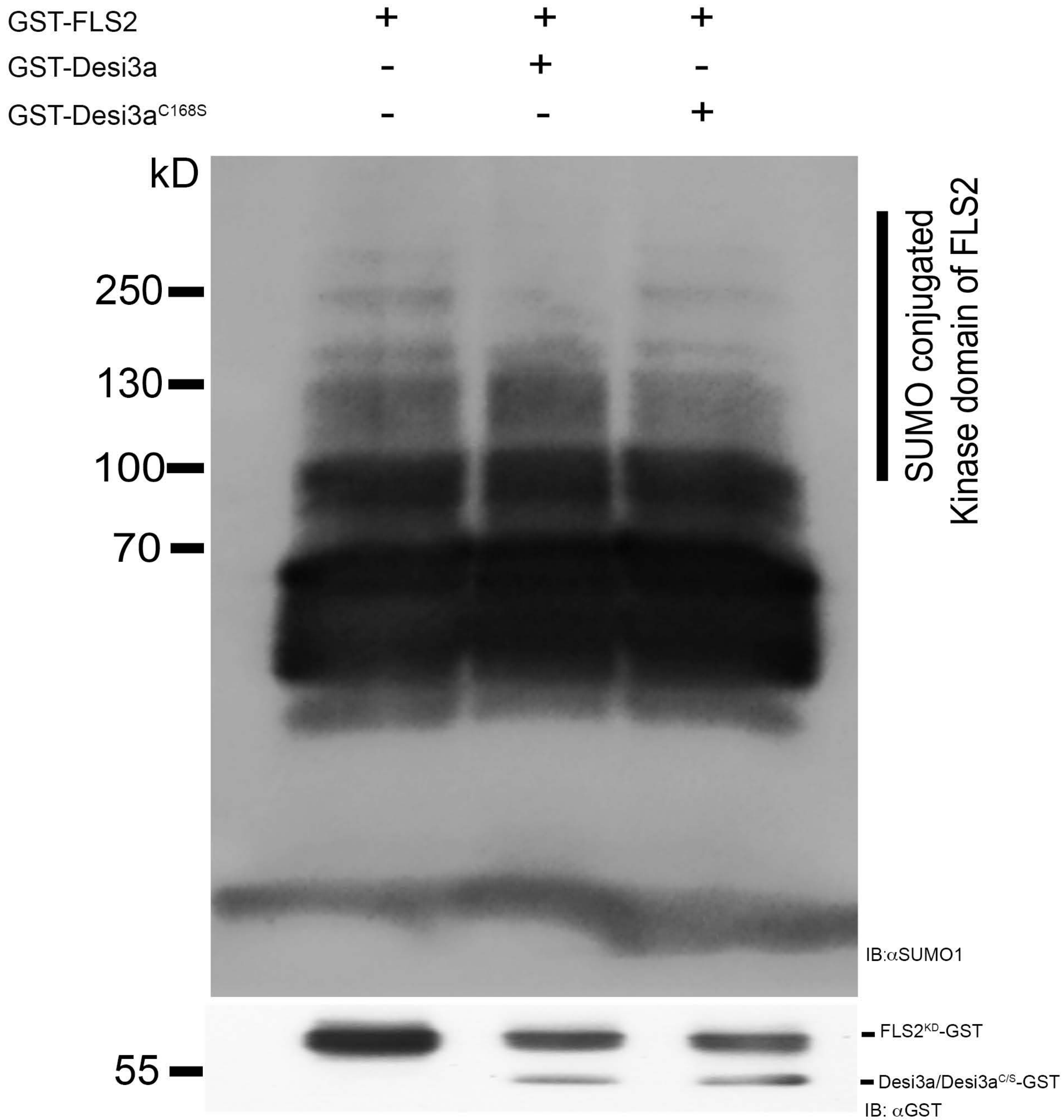

Supplementary Figure 13

FLS2 SUMO conjugates are cleaved by Desi3a protease. *In vitro* deSUMOylation of SUMOylated GST-FLS2 kinase domain proteins after incubation with GST-Desi3a or GST-Desi3a^{C168S} recombinant proteins.

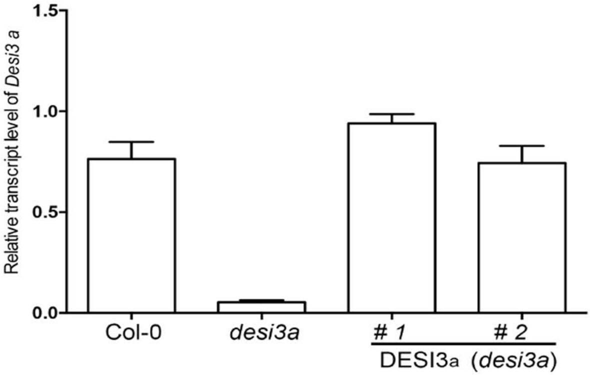

Supplementary Figure 14

Expression analysis of complemented lines for Desi3a in *desi3a-1* mutant background. The expression levels of Desi3a in Col-0, desi3a and two different complemented lines by real-time PCR analysis. The experiment was replicated three times and data represent mean value ± SD (N = 3).

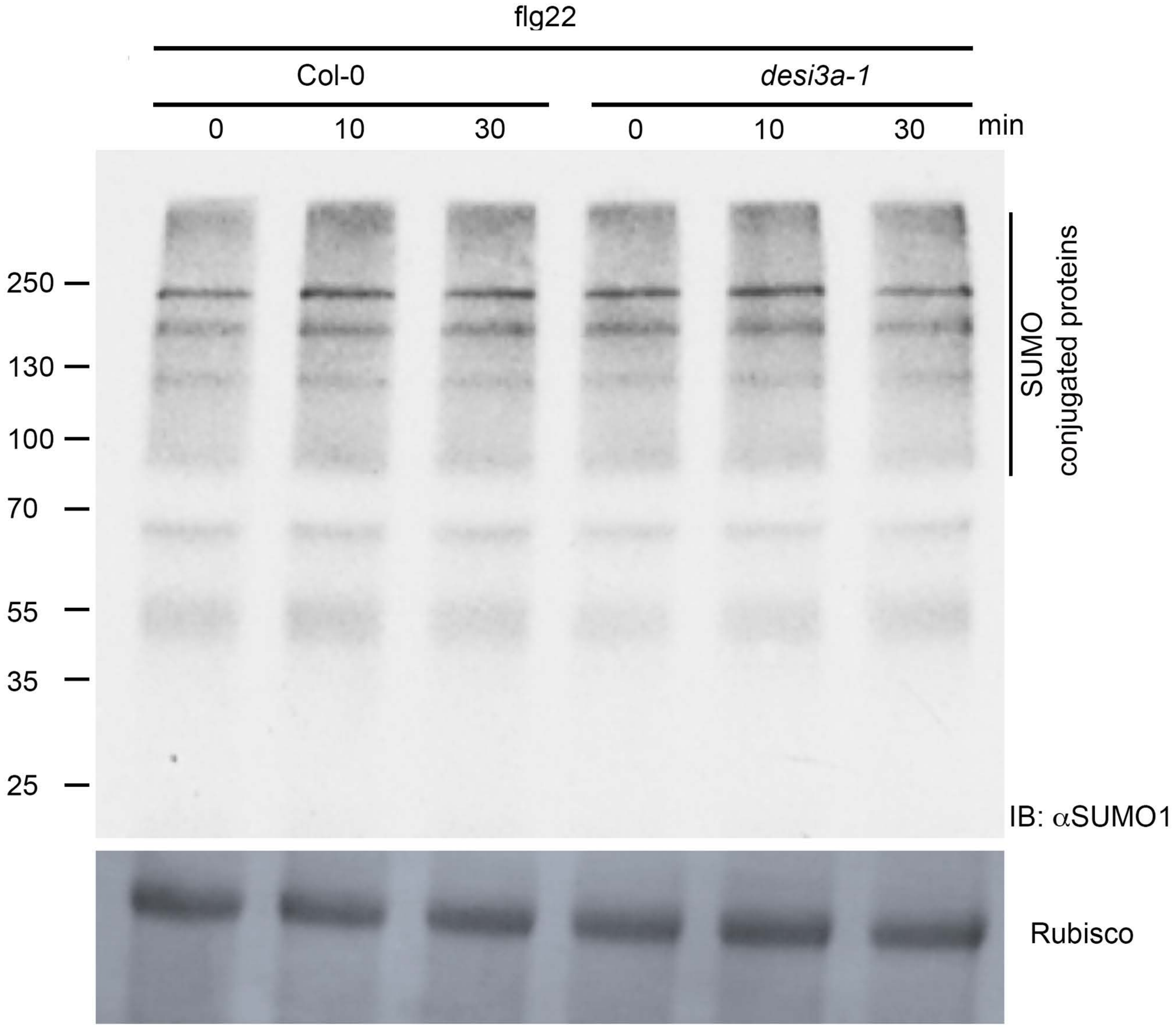

Supplementary Figure 15
SUMO conjugates accumulation was similar in *desi3a-1* and Col-0 plants after flg22 treatment. Homozygous desi3a-1 seedlings accumulate more SUMO conjugates during flg22 (1μM) treatment. Seven-day-old wild-type Col-0, and *desi3a-1* seedlings were either treated with water or flg22 for 10min and 30min. Total protein extracts were separated by SDS-PAGE and subjected to immunoblot analysis with anti-SUMO1 antibody. SUMO conjugates are indicated by a black solid line.

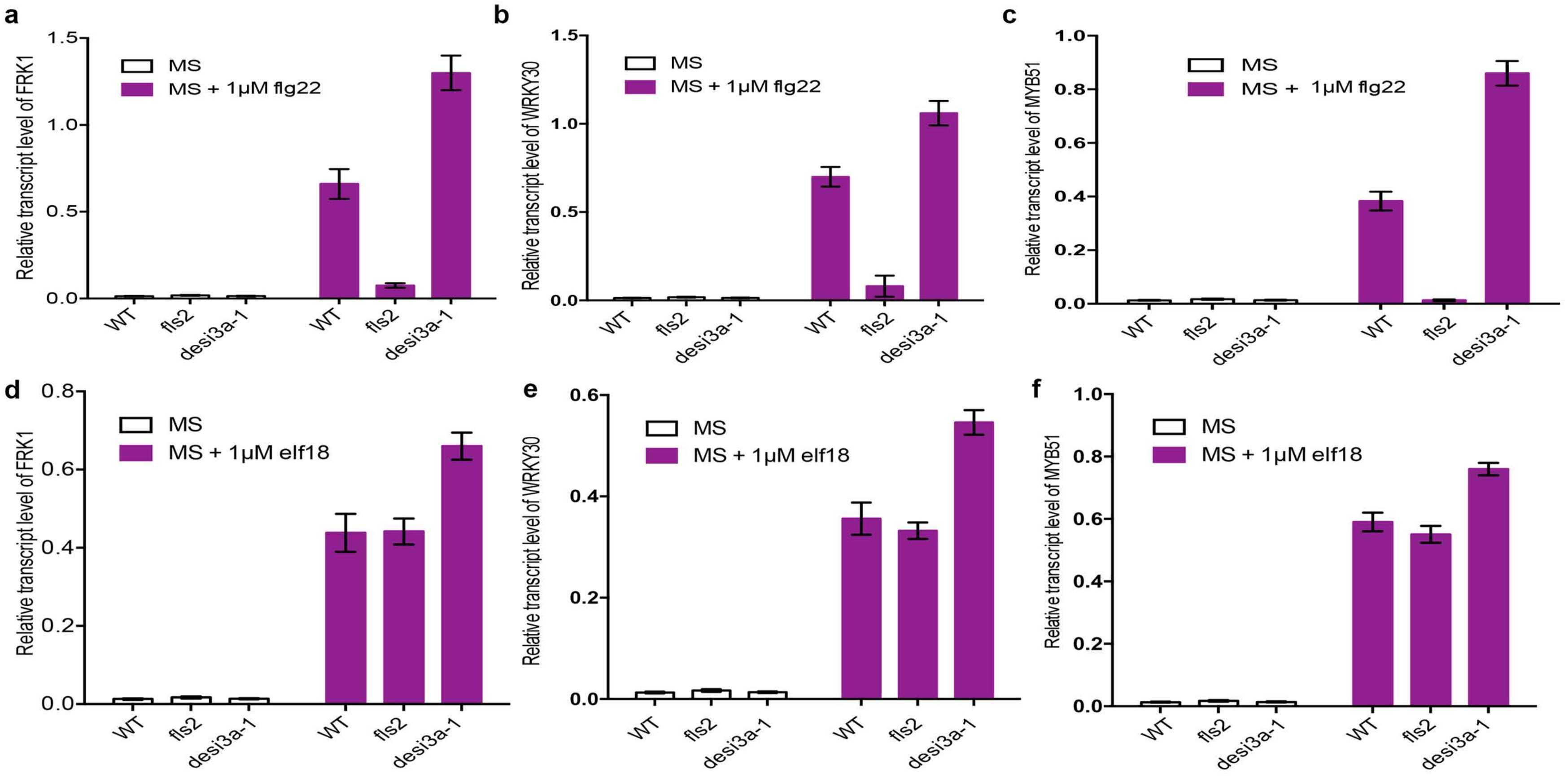

Supplementary Figure 16

Quantitative RT-PCR analysis to detect FRK1, WRKY30 and MYB51 transcript abundance in the various mutants. **(a)** RT-PCR analysis of transcripts from 10-day-old Col-0, *fls2* and *desi3a-1* mutant lines. Total RNA was isolated from 10-day old Col-0 and the homozygous *fls2* and *desi3a-1* seedlings treated with flg22. **(b)** Quantitative RT-PCR analysis to detect FRK1, WRKY30 and MYB51 transcript abundance in the indicated mutants after elf18 treatment. Actin primers were used as controls to determine total cDNA levels.

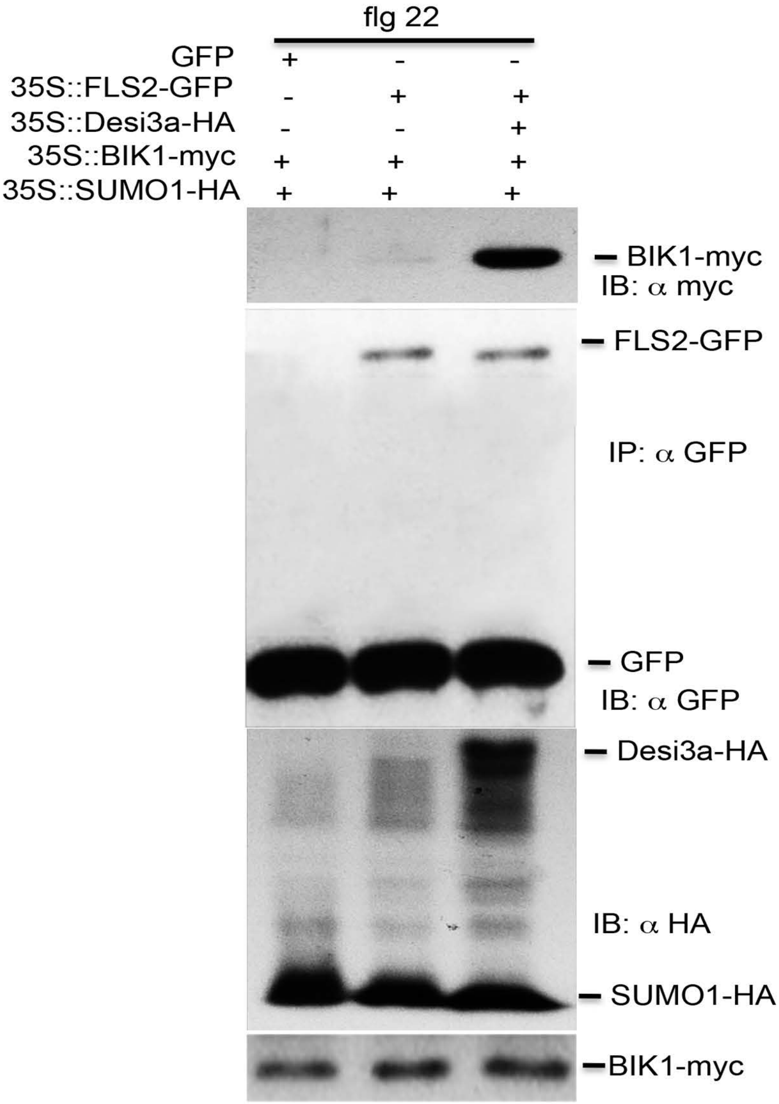

Supplementary Figure 17

Desi3a regulates FLS2-BIK1 interaction by deSUMOylating FLS2. FLS2-GFP, BIK1-myc and SUMO1-HA were co-expressed in *N. benthamiana* leaves either without or with HA-tagged Desi3a in the presence of 1 μM flg22 (10 min). Immunoprecipitation assays to pull down FLS2-GFP was done with anti-GFP antibody beads (IP: α GFP). Immunoblots were probed with anti-GFP (IB: α GFP) for FLS2-GFP, anti-c-myc (IB: α myc) for BIK1-myc and anti-HA (IB: α HA) for SUMO1-HA and Desi3a-HA. BIK1 protein levels were ascertained in input lanes by probing with anti-myc antibodies to detect BIK1-myc in total protein extracts.

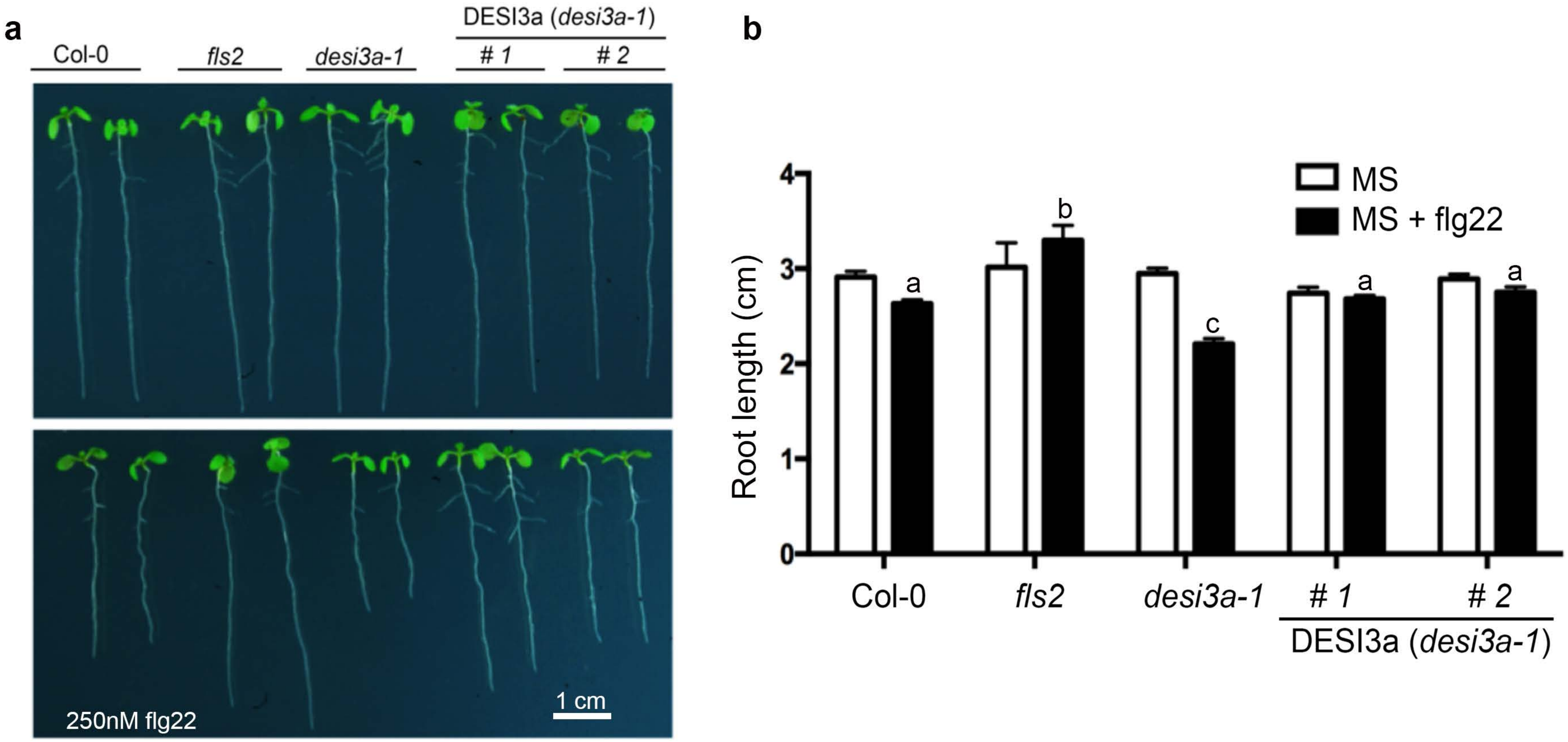

Supplementary Figure 18

(a) *desi3a-1* sensitivity to flg22 mediated root growth inhibition is reversed by expressing Desi3a. Image of representative Arabidopsis seedlings of Col-0, *desi3a-1*, Desi3A complementation lines #1 and #2 after 5 days on 0nM and 250nM flg22-containing medium. **(b)** Quantification of the root length of the different genotypes.

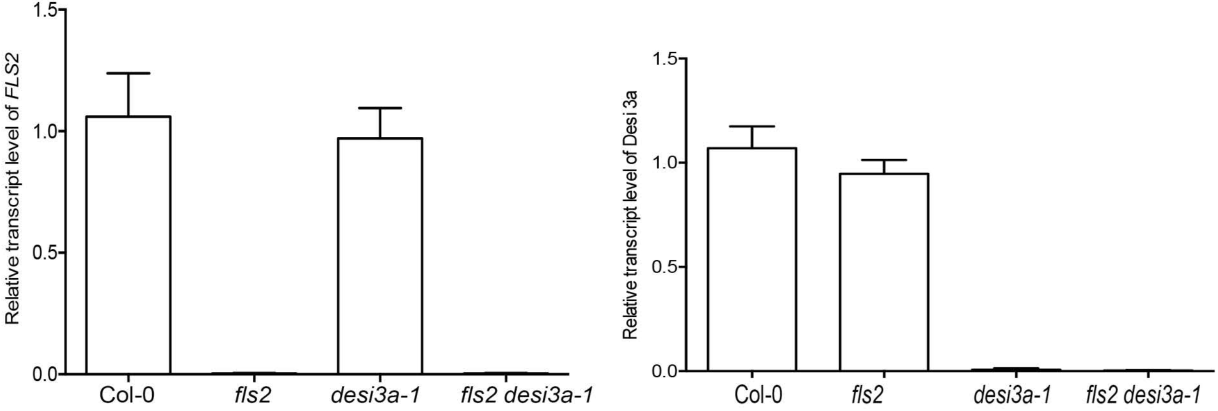

Supplementary Figure 19

RT-PCR analysis of transcripts from 10-day old Col-0, the homozygous *fls2* and *desi3a-1* mutant lines used for producing the double mutant, and the *fls2 desi3a-1*double mutant. Quantitative RT-PCR analysis to detect FLS2 and Desi3a transcript abundance in the double mutants. Total RNA was isolated from 10-day old Col-0, the homozygous *fls2*, *desi3a-1* and *fls2 desi3a-1* mutant plants. Actin primers were used as controls to determine total cDNA levels. . Data present mean ± SD from three independent replicates (N=3).

Supplementary Table 1: List of peptides identified in MS

S.No.	Gene ID	Name	% Coverage	Highest Confidence Peptides Identified
1	At2g30950	FTSH2 (ATP-dependent zinc metalloprotease)	6.33	GVLLIGPPGTGK; QVSVDVPDVK
2	At1g42970	GAPB (Glyceraldehyde-3-phosphate dehydrogenase)	26.85	AAALNIVPTSTGAAK; AVSLVLPQLK
3	At3g60750	TKL-1 (Transketolase-1)	19.03	AAVETVEPTTDSSIVDK; AFGDFQK
4	At2g21330	FBA1 (Fructose-bisphosphate aldolase 1)	28.57	AAQDILLAR; ANSLAQLGK
5	At4g13940	SAHH1 (Adenosylhomocysteinase 1)	18.76	AGIIVLAEGR; ATDVMIAGK
6	At3g45140	LOX2 (Lipoxygenase 2)	10.27	ANIEQEGNTVK; ELNNTTLYASR
7	At1g56070	LOS1 (Elongation factor 2)	9.13	EGPLAEENMR; FSVSPVVR
8	AtCg00340	psaB (Photosystem I P700 chlorophyll a apoprotein A2)	7.76	DKPVALSIVQAR; DYNPEQNEDNVLAR
9	At5g50920	CLPC1 (Chaperone protein ClpC1)	8.18	AEVSAIQAK; AIDLIDEAGSR
10	At5g38410	RBCS-3B (Ribulose bisphosphate carboxylase small chain 3B)	22.65	EHGNTPGYYDGR; EYPGAFIR
11	At4g24280	HSP70-6 (Heat shock 70 kDa protein 6)	7.93	NQADSVVYQTEK; TTPSVVAYTK
12	At3g45980	H2B (Histone H2B.3)	19.87	LVLPGELAK; QVHPDIGISSK
13	At3g53420	PIP2-1 (Aquaporin PIP2-1)	11.85	AFQSSYYTR; AKDVEAVPGEGFQTR
14	At1g75780	TUBB1 (Tubulin beta-5 chain)	6.68	FPGQLNSDLR; INVYYNEASGGR
15	At5g46330	FLS2 (Flagellin-sensitive 2)	23.96	SSESSLPDLDSALK; PANILLDSDRVAHVSDFGTAR
16	At2g17360	RPS4A (40S ribosomal protein S4-1)	10.34	GIPYLNTYDGR; LGNVYTIGK
17	At2g05710	ACO3 (Aconitate hydratase 3)	4.44	TSLAPGSGVVTK; DFNSYGSR
18	At1g04410	MDH1 (Malate dehydrogenase 1)	7.83	LSVPVSDVK; EFAPSIPEK
19	At5g47210	RGGC (RGG repeats nuclear RNA binding protein C)	10.92	AAAAVQPPK; FPTKPAPPSQAVR
20	At1g20020	LFNR2 (Ferredoxin--NADP reductase, leaf isozyme 2)	9.48	LVYTNDQGETVK; VDYAISR
21	At3g01500	BCA1 (Beta carbonic anhydrase 1)	12.39	AFDPVETIK; GGYYDFVK
22	At4g20260	PCAP1 (Plasma membrane-associated cation-binding protein 1)	10.22	VSVFLPEEVK; VVETYEATSAEVK
23	At1g31340	RUB1 (Ubiquitin-NEDD8-like protein)	17.87	IQDKEGIPPDQQR; TLADYNIQK
24	At1g49760	PAB8 (Polyadenylate-binding protein 8)	2.98	LANAAPEQQR; VAVDPSGQSK
25	At4g26840	SUMO1 (Small Ubiquitin-like Modifier 1)	10.23	GQDGNEVFFR
26	At3g51800	EBP1 (ERBB-3 BINDING PROTEIN 1)	6.38	NTDVTEAIQK; SAAEIVNK
27	At1g12840	VHA-C (V-type proton ATPase subunit C)	6.65	VAEYNNIR; LVQDQESLR
28	At4g09000	GRF1 (14-3-3-like protein GF14 chi)	10.06	IISSIEQKEESR; IISSIEQK
29	At5g67500	VDAC2 (Mitochondrial outer membrane protein porin 2)	3.62	TAAVGEVYR
30	At5g42270	FTSH5 (ATP-dependent zinc metalloprotease)	5.39	LESGLYSR; YSEFLNAVK

A list of proteins that match the peptides identified from Mass Spectrometry analysis using Strep-tagged AtSUMO1. The AGI codes, percentage coverage and high confidence peptides identified for each protein are included in the table.

Supplementary Table 2: List of the constructs used for the study

Genes Used	Constructs	Vectors	Experiments
FLS2	FLS2-GFP	pEG103	Co-IP/ Confocal
$FLS2^{K/R}$	$FLS2^{K/R}$-GFP	pEG103	Co-IP/ Confocal
*pro*FLS2::FLS2	FLS2-GFP	pMDC109	Generation of transgenic plants
*pro*FLS2::$FLS2^{K/R}$	$FLS2\text{-}GFP^{K/R}$	pMDC109	Generation of transgenic plants
BAK1	BAK1-myc	pGWB17	Co-IP
BIK1	BIK1-myc	pGWB17	Co-IP
SCE1 (E2)	HA-SCE1	pEG201	Co-IP
Desi3a	HA-Desi3a	pEG201	Co-IP/ Generation of transgenic plants
Desi3a	mCherry-Desi3a	pGWB15	Confocal
Desi3a	GST-Desi3a	pDEST15	Isopeptide-cleaved SUMO chains
Desi3a C168S	GST-Desi3a C168S	pDEST15	Isopeptide-cleaved SUMO chains
SUMO1	His-SUMO1	pDEST17	Isopeptide-cleaved SUMO chains
$FLS2^{KD}$	GST-$FLS2^{KD}$	pDEST15	In vitro SUMOylation and deSUMOylation
Strep-$SUMO1^{H89R}$	Strep-$SUMO1^{H89R}$ (with stop codon)	pEG301	Generation of transgenic plants for mass spectrometry
At2g25190	HA-At2g25190	pEG201	Co-IP
At2g25190	GFP- At2g25190	pEG104	Confocal

Supplementary Table 3: List of primers used for the study

Genes	Forward Primer	Reverse Primer	Function
*pro*FLS2::FLS2	5’ CACCATGAAGTTACTCTCAAAG 3’	5’ AACTTCTCGATCCTCGTTAC 3’	Cloning
BAK1	5’ CACCATGGAACGAAGATTAATGA 3’	5’ TCTTGGACCCGAGGGGTATT 3’	Cloning
BIK1	5’ CACCATGGGTTCTTGCTTCAGTTC 3’	5’ CACAAGGTGCCTGCCAAAAG 3’	Cloning
Desi3a	5’ CACCATGTTGAACGGAAAAGAA 3’	5’ CCTTTCTTTCAAGGAGCTGCT 3’	Cloning
SCE1	5’ CACCATGGCTAGTG GAATC 3’	5’ TTA GACAAGAGCA GGATAC 3’	Cloning
$FLS2^{KD}$	5’ CACCATGGAAAATTCATCA 3’	5’ CTAAACTTCTCGATCCTCGTT 3’	Cloning
FLS2 K1120R	5’ TGTGAGACAGGAAGAGGC 3’	5’ GCCTCTTCCTGTCTCAGA 3’	Site-directed mutagenesis
WRKY30	5’ AGAACGCTGGACGATGG 3’	5’ TGTTCTTGGGTCTGGTTC 3’	Q-PCR
MYB51	5’ ACGTGTTCTTCGTCCACG 3’	5’ TAGACCGGCGTCACATC 3’	Q-PCR
FRK1	5’ GCCAACGGAGACATTAGAG 3’	5' CCATAACGACCTGACTCATC 3'	Q-PCR
Desi3a	5’ TCAGTCTCAGGTGGTTCACAG 3’	5’ GCGACTCTGACCAACCTTTCT 3’	Q-PCR
FLS2	5’ CAGTCCTAACGGTGGGGTTC 3’	5’ GGTTGTGAGACAGGTCCAGG 3’	Q-PCR
ACT7	5’ CCATCGCTCATCGGAATGGA 3’	5’ TGGAACCACCACTGAGAACG 3’	Q-PCR
At2g25190	5’ CACCATGTTGTGCTTCAAAGGCTC 3’	5’ TCAAGTCTTGATGCTTACGC 3’	Cloning
Desi3a	5’ GTTCCGAAATCGGCAAAAT 3’	5’ ATTTTGCCGATTTCGGAAC 3’	Genotyping
FLS2	5’AGGGTTTGCGTGGGAAAG 3’	5’GACATGGTTTTCTATCAGTCTA 3’	Genotyping

(a)

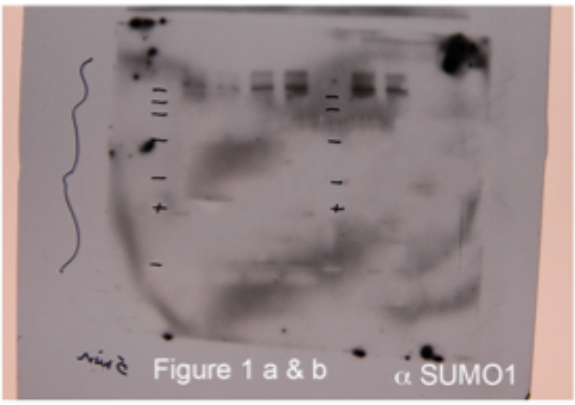

(b)

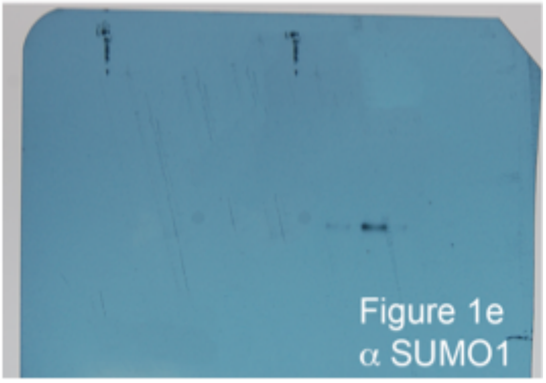

Supplementary Figure 20
The blots are uncropped versions of the final blots shown **(a)** in Fig. 1a and b and **(b)** in Fig. 1e. Both the blots were immunoblotted with αSUMO1 antibody.

(a)

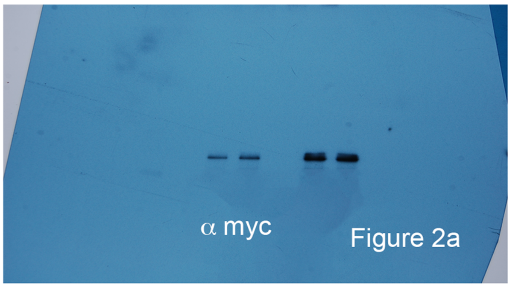

(b)

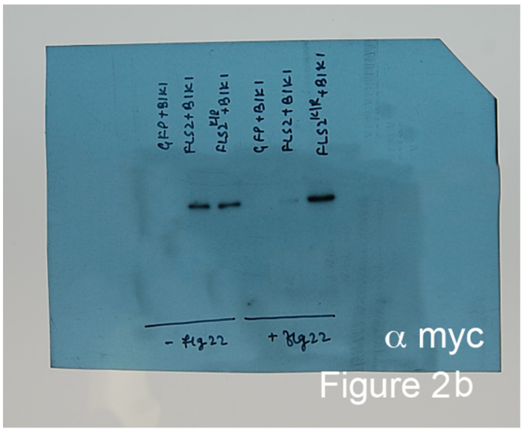

(c)

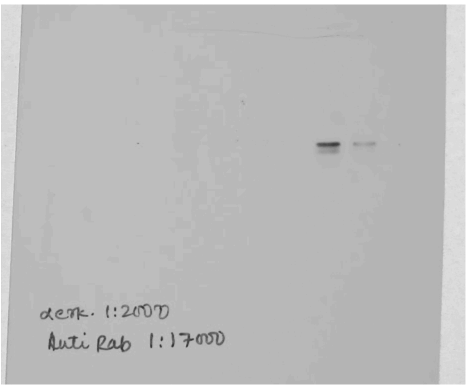

Supplementary Figure 21

The blots are uncropped versions in support of the final blots shown **(a)** in Fig. 2a, **(b)** in Fig. 2b and **(c)** in Fig. 2d. The blots in (a) and (b) were immunoblotted with α-c-myc antibody, whereas (c) was immunoblotted with αpErk1/2 antibody.

(a)

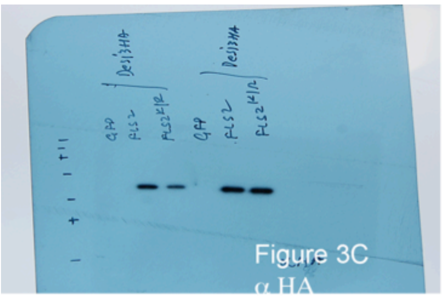

(b)

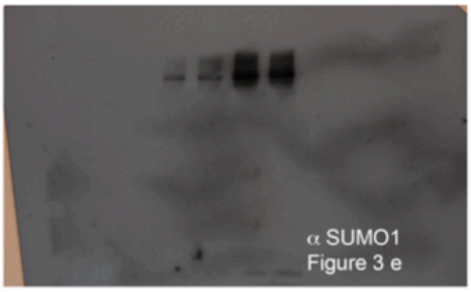

(c)

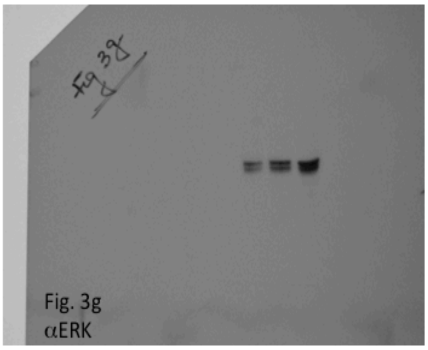

(d)

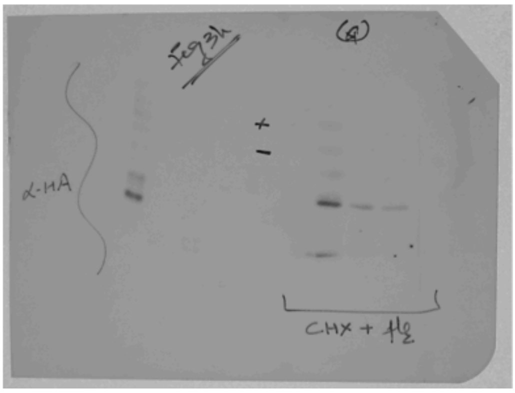

Supplementary Figure 22

The blots are uncropped versions in support of the final blots shown **(a)** in Fig. 3c, **(b)** in Fig. 3e, **(c)** in Fig. 3g and **(d)** in Fig. 3h. The blots in (a) and (d) were probed with αHA antibody, in (b) with αSUMO1 antibody and in (c) with αpErk1/2 antibody.

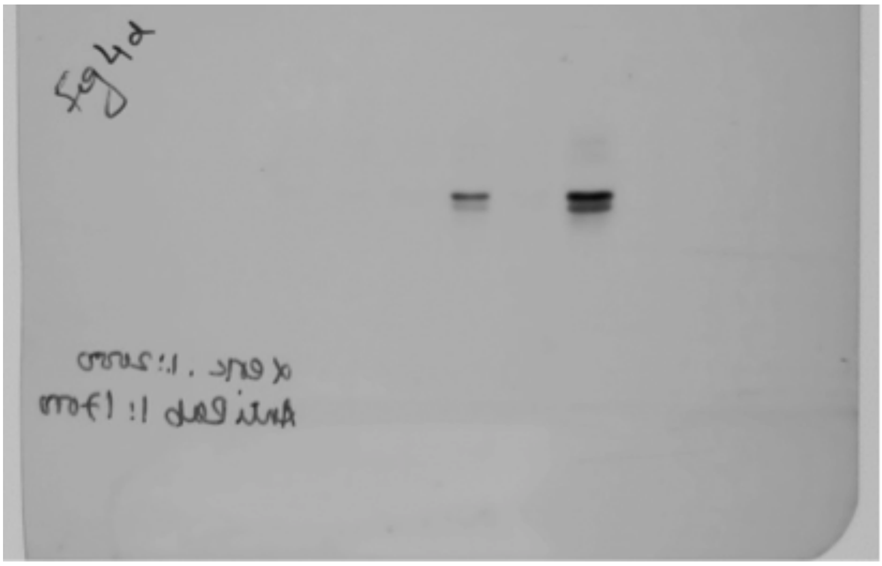

Supplementary Figure 23
The blot is an uncropped version in support of the final blots shown in Fig. 4d.
The blot was immunoblotted with αpErk1/2 antibody.

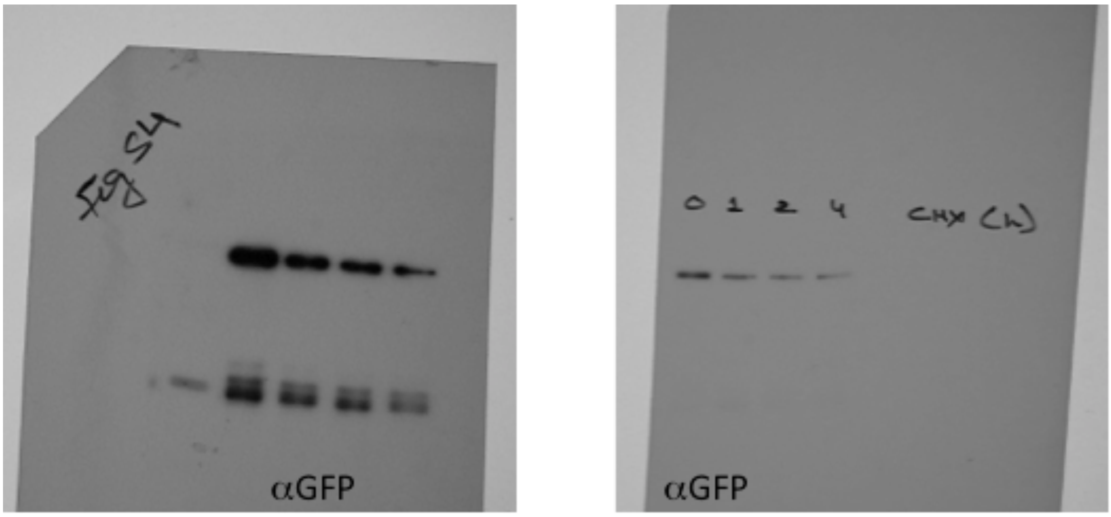

Supplementary Figure 24
The blots are uncropped versions in support of the final blots shown in Supplementary Figure 4. The blot was immunoblotted with αGFP antibody.

(a) **(b)**

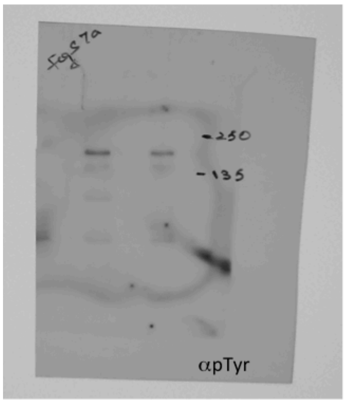

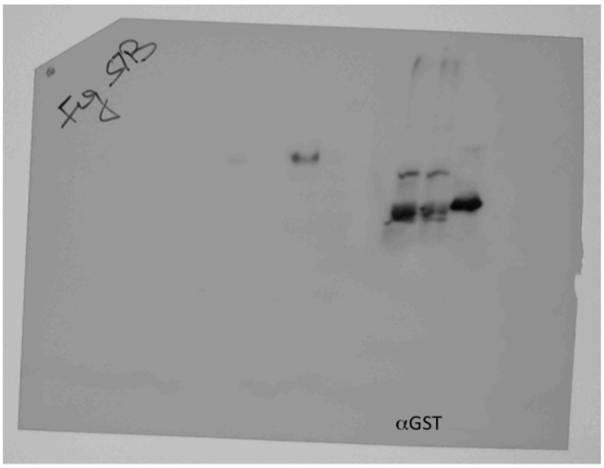

Supplementary Figure 25
The blots are uncropped versions in support of the final blots shown in Supplementary Figure 7. The blot in (a) was immunoblotted with αpTyr, while the blot in (b) was immunoblotted with αGST antibody.

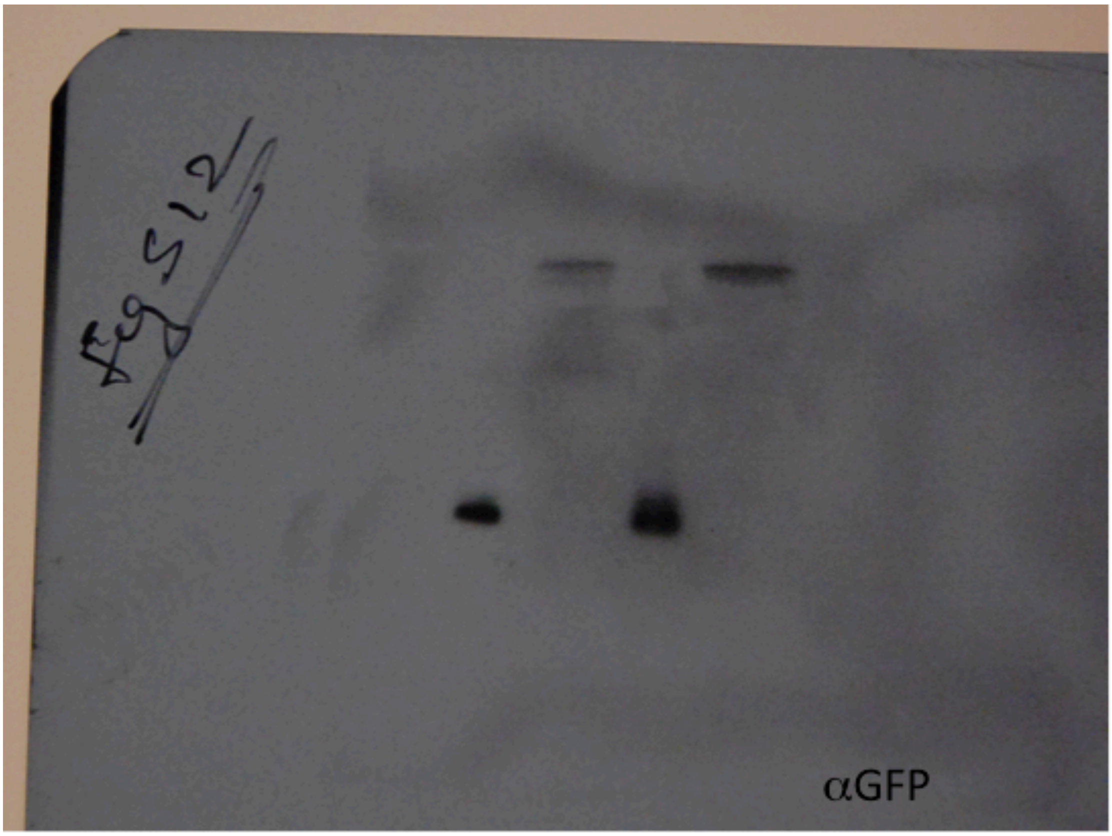

Supplementary Figure 26
The blot is an uncropped version in support of the final blot shown in Supplementary Figure 12. The blot was immunoblotted with αGFP antibody.
